# DFT studies of solvent effect in hydrogen abstraction reactions from different allyl-type monomers with benzoyl radical

**DOI:** 10.1186/s13065-023-01027-9

**Published:** 2023-09-12

**Authors:** Xiaotian Zhao, YaMing Li, Shibo Lin, Chun Liu, Xirui Guo, Xuanhao Li, Lihui He, Xi Chen, Guodong Ye

**Affiliations:** 1 Department of Pharmacy, Chengdu Second Peoples Hospital, Chengdu, 610017 People’s Republic of China; 2 Department of Stomatology, Chengdu Second Peoples Hospital, Chengdu, 610017 People’s Republic of China; 3 Guangzhou Municipal and Guangdong Provincial Key Laboratory of Molecular Target and Clinical Pharmacology, the NMPA and State Key Laboratory of Respiratory Disease, School of Pharmaceutical Sciences and the Fifth Affiliated Hospital, Guangzhou Medical University, Guangzhou, 511436 China

**Keywords:** Allyl, Solvent effect, DFT calculations, Hydrogen abstraction reaction, Transition state

## Abstract

Inert allyl-type monomers have been widely documented due to reduce degradation chain transfer. Recently, we and others discovered that the [3 + 2] cyclization reaction process by a photo-driven radical reaction, which can accelerate the polymerization. It was discovered that allyl ether monomers had much higher reactivity than other allyl monomers in the suspension photopolymerization initiated by Type I photoinitiator. Since the hydrogen abstraction reaction (HAR) is the initial step of cyclization, and in order to clarify the influence of solvents effect, three allyl-type monomers were employed, containing “O”, “N” and “S” atom as hydrogen donors. The benzoyl radical obtained from cleavage of photoinitiator was chosen as hydrogen acceptors. We explored the hydrogen abstraction reaction in different solvents (methanol, water and DMSO) by quantum chemistry for geometry and energy. An investigation was undertaken regarding the structural orbital by electrostatic potential (ESP) and topological analysis (ELF and LOL). The findings were also combined with the distortion model and transition state theory. We obtained the molecular interactions used independent gradient method in the Hirshfeld molecular density partition (IGMH). The Eckart’s correction allowed to examine the driving factors of the hydrogen abstraction reaction tunnels and these reactions constant rates are determined in the range of 500–2500 K depending on the modified Arrhenius form in different solvents effect. Our results can provide an answer for the different reactivities.

**Supplementary Information:**

The online version contains supplementary material available at 10.1186/s13065-023-01027-9.

## Introduction

Over the past few years, interest has increased in allylic compounds fields due to their exceptional physical and electrical properties [1]. Allyl substances are characterized by the occurrence of the allyl part CH_2_=CH–CH_2_–X–R (X = CH_2_, N, O, CONH, …etc.). They are applied to enhance thermal consistency and wear on other materials [2] in coating and copolymers. Reactions to thiol-ene are founded on CH_2_=CH– using initiators and Fenton reactions focus primarily on adjacent CH_2_ applying cobalt driers [3, 4]. Polymers are also manufactured through the integration of polymerization and copolymer applications in the mechanical industries [5].

In our past work [6, 7], the embolismic agent was synthesized based on allylic monomers have a novel method in interventional treatment and the allylic polymers has given rise to interest in recent years [8].

In the past, direct homo-polymerization of allyl-type compounds was reported as notoriously difficult and yield polymers at low yield and molecular weight initiated by thermal initiators [9]. The photo-polymerization method was used to polymer allyl multi-ether monomers to get the embolismic agent through a easily cycling reaction mechanism [3 + 2] called photo-driven radical mediation (PRMC) [10]. The initial reaction is a process of abstracting hydrogen as hydrogen migration of the α-methylene group of the allylic group to the stage of triplet or photo-initiator fragments, producing primary allyl radicals, then initiates the [3 + 2] cycling reaction [11].

The hydrogen abstraction reaction (HAR) is major initiation reactions producing primary allyl radicals and leading to chain propagation without degradation chain transfer [12]. It is easily for these reactions to polymerize with free radicals (meth)acrylates or photo-induced auto-oxidation of alkyd resins [13, 14], which played a major role in the first stage [15]. HAR occurs between H-donors and H-acceptors. H-acceptors may be triplet state compounds or radicals. H-donors are usually polar hydrogen such as hydrogen atoms in the alcohol, etc. However, more and more alkanes’ C–H with *sp*^3^ hybridization hydrogen get increasingly attention, e.g. C–H of methene in polyunsaturated fatty acids or cyclic acetals [16]. Though HAR studied in many literatures, experimental and theoretical investigations of kinetic parameters of hydrogen extraction from allyl monomers in solvent effects do not exist. Solubility is a key thermal dynamic characteristic for chemicals and application, since it contributes to purifying organic compounds and design of synthetic route in future.

**Scheme 1:**
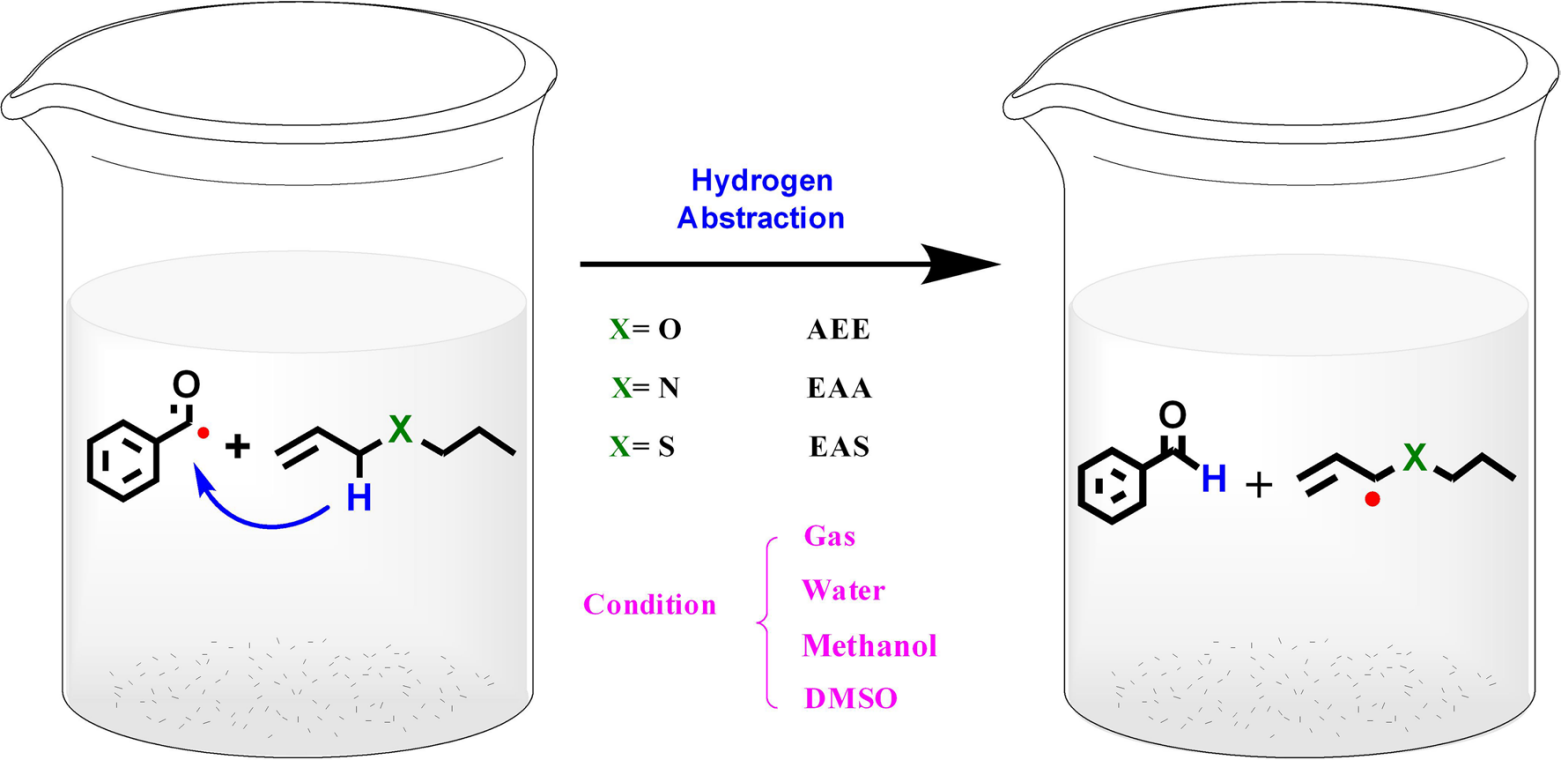
The diagram of excited BR as hydrogen acceptors reacting with C–H donors from allyl-type monomer in different solvents

In our study model, three allyl monomers were used as potential donors and benzoyl radicals (BR) as acceptors in order to select a click compound from three allyl monomers after screening. The structure of molecules in our model is shown in Scheme 1. The BR can be obtained from the Norrish I cleavage of HMPP (2-Hydroxy-2-methylpropiophenone) photoinitiators (Darocur 1173). We will discuss solvent effects based on the structural parameters and reaction properties by high-precision quantum chemical method.

In addition, the three allylic monomers geometry in solutions, including acceptors' conformation and the coordinates of the transition state (TS), as well as a solvation investigation of thermodynamic parameters and kinetic descriptors by vary from different temperature effect.

We examined computed orbital analysis, thermodynamic parameters, and electronic feature [17–20]. Aiming to improve the polymerization effectively in different solvents in next step, the HAR study is a crucial step in the initial stage. Moreover, the IGMH method [21] is to provide insight into the inter-molecular interactions between certain groups of atoms in different solvents. Furthermore, our goal is to offer precise parameters of kinetic and facilitate polymer development for modeling the effect of the solvent.

## Computational details

The entire series of calculations were evaluated by density functional theory [22] (DFT) by Gaussian 16 program [23] and GaussView 6.0. The solvent environment was represented using the implicit solvent model (SMD) based on density [24]. The three different monomers, the allyl ethyl ether monomer (AEE), ethyl allyl amine monomer (EAA) and ethyl allyl sulfide monomer (EAS), were selected to represent allyl type compounds. In M06-2X functional and 6–311++ g(d,p) level [25] was employed to evaluate the geometry optimization, the energies value and barrier heights, scaled by a zero energy scale factor (ZPE) of 0.97 [26]. Specially, the def2tzvp basis set was chosen to get specific HOMO–LUMO orbitals. The vibration frequencies of the optimized structures are calculated without imaginary frequencies of the stable structures. Besides, only one imaginary TS frequency was existed [27]. We implemented intrinsic reaction coordinate [28] (IRC) calculations to verify that the TS connects the two right stationary points. Immediately, we optimized the product and reactant structure separately for next research.

The electronic feature, including electrostatic potential (ESP) [20], electron localization function [18] (ELF) and localized electron locator [19] (LOL) of title molecules were gotten by using Multiwfn 3.8 [29] and VMD 1.9 [30] software in gas phase and other solvents. The IGMH is also got by Multiwfn 3.8 and VMD 1.9 software. To immediately visualize electron density, different color code was used: red stands for a powerful repulsion force; van der Waals was represented by green, and blue color for a highly attractive force. Using KiSThelP 2016 [31], the values of *k* and tunneling factors (*κ*(T)) were calculations. We also carried out the k of these groups, which have a relationship with temperature. The high-temperature-limit k of hydrogen reactions value by used the transition state theory (TST) way. Using TST method in different solvents effect with Eckart’s tunneling factors was used to acquire high-temperature-limit of rate constants about 12 reactions. A modified Arrhenius formula was used to represent each rate constant and the temperature varying from 500 to 2500 K treated with TST method. More calculations data are displayed in the Additional file 1.

## Result and discussion

### Orbital analysis of allyl-type monomers

**Fig. 1:**
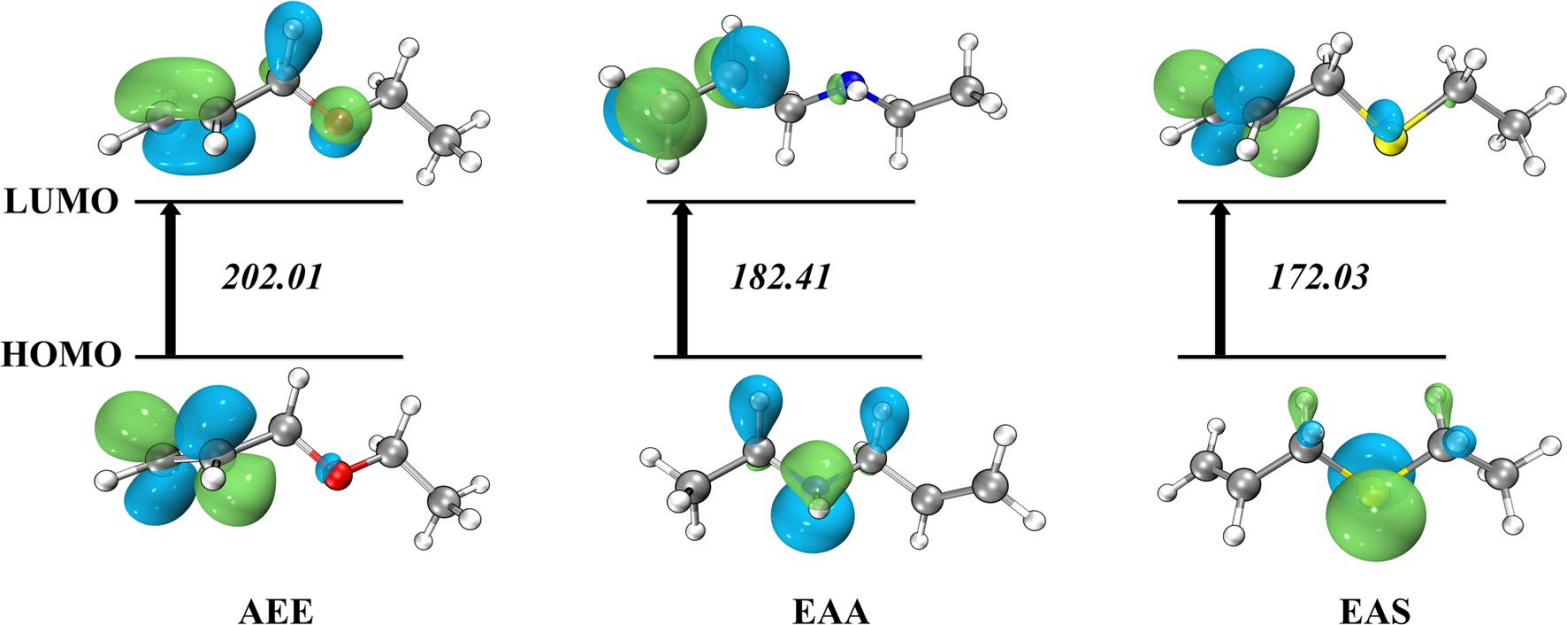
The HOMO and LUMO values of the AEE, EAA and EAS using M06-2X/def2tzvp basis set in gas phase (Unit: kcal/mol)

It is a useful practical model for describing chemical property and bioactivity [32] by frontier molecular orbital. This theory makes it possible to identify electrophilic attacks and nucleophile responsible for forming hydrogen bond interactions [33]. For the highest occupied molecular orbital (HOMO) and the lowest unoccupied molecular orbital (LUMO) energies, these are significant quantitative mechanical descriptors to depict the FMO characteristic. Figure 1 shows the HOMO–LUMO energy gap values of the three monomers. As is shown above, the HOMO (24) and LUMO (25) orbitals contributed significantly to the AEE energy levels obtained of 202.01 kcal/mol, and HOMO and LUMO energy value at − 8.77 and − 0.01 eV from Additional file 1: Table S1, respectively. Beyond that, there is obviously to find that the electrons are entirely localized on the “N” and “S” atom from EAA and EAS, separately. When the electrons enter into LUMO orbital, the electrons will totally gather at alkene bond corresponding the π → π*. However, the mainly electron cloud is localized on the carbon–carbon bond at the HOMO orbital from AEE molecular. The more information detail could be found in Additional file 1: Table S1.

The kinetic energy is implied by the FMO energy gap, which also predicts chemical reactivity [32]. Soft organic molecules are defined as having a tiny FMO energy gap and a high level of chemical reactivity[34].The EAS shows the lowest value at 172.03 kcal/mol which indicates the allyl-type monomers contain sulfur atoms have more intensely reacting ability in theory. Compared to the computed results of solvent effect shows that the solvent increases the energy gap and the AEE of HOMO–LUMO energy gap value at 208.11 kcal/mol was the supreme energy in water solvent in Additional file 1: Table S1.

Understanding the chemical characteristics of different compounds through parameters [35]. There are so much parameters (Additional file 1: Table S2) to describe the molecular characteristic, and we obtained these parameters using M06-2X/6-311G++ basis set. Chemical hardness (η) is a descriptor of a compound's thermodynamic stability. Electronegativity (χ) is a molecule's or functional group's ability to draw electrons to itself [36]. The global electrophilicity index (ω) illustrates the stability of the system following the additional electrons it receives from the environment. Namely, the electrophilic index (ω) determines whether a molecule will behave as an electrophile or a nucleophile when the molecules react. More electrophilic is the value that is greater and vice versa [37]. According to the Additional file 1: Table S2, it showed the electrophilic index (ω) value of the AEE is the highest, indicating that the AEE is more electrophilic. That is, it demonstrates that as an electrophilic value, AEE is more capable of chemically interacting with other compounds in gas phase. Compared the chemical hardness (η), we obtained the maximum value for AEE molecular in water solvent and solvent effect could improve molecular stable ability. The more detail data were displayed from Additional file 1: Table S2.

### Electronic features of allyl-type monomers

**Fig. 2:**
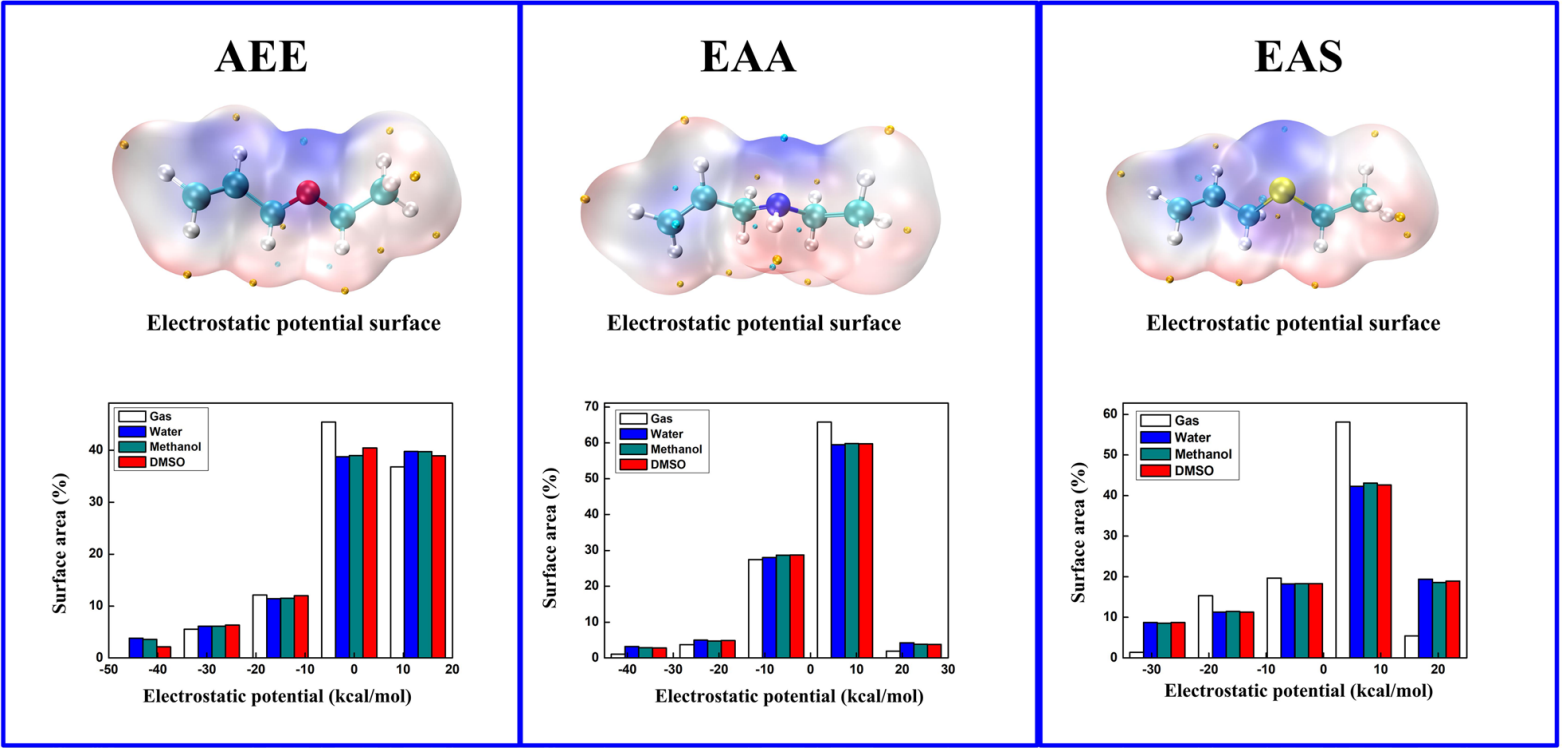
The three title molecular vdW surfaces were mapped by ESP: AEE, EAA and EAS in gas phase. The value of molecule’s surface local minima and maxima of ESP are shown as yellow and cyan spheres, respectively (Unit: kcal/mol). The area percent within each ESP range of different solvents in below part

Electrostatic potential (ESP) surface used to study reactivity and anticipating the interaction between molecules [38, 39]. Comprehensive investigation of ESP of AEE, EAA and EAS must be very beneficial for deeper comprehension of the TS interaction in next part. The various ESP values are represented by distinct colors. According to the Fig. 2, the cyan and yellow balls, respectively, reflected the negative and positive of ESP. An electrophilicity is described in red area, whereas a nucleophilic site is depicted in blue, which explains the large positive charges and highly negative charges of the molecule’s surface, respectively. The calculated results demonstrate that the potential negative region is near “O”, “N”, “S” atom and the –O–CH_2_ –CH=CH_2_ group has a value for ESP reach − 46.35 kcal/mol in water solvent in Additional file 1: Table S3. Besides, different ESP range surface areas in various solvents are measured as shown in Fig. 2 below part, it indicated that the large part of vdW surface of AEE have the lowest value range from − 1 to + 13 kcal/mol, the most part of the other compound’s vdW surface value above the 0 kcal/mol. Compared to gas phase, the results of ESP research shows that liquid influence grows the nucleophilic and electrophilic. The AEE monomers with higher negative ESP had a greater electrophile attraction and are therefore more likely to be the reactive site.

By using atoms in molecular theory [40] topological analysis was performed to research. The ELF topological analysis (electrons are highly localized) denoted by (r) and LOL, contributed by ƞ(r) were carried out. The chemical formulas of ELF and LOL rely on the density of kinetic energy D(r) caused by the Thomas–Fermi kinetic energy density D_0_(r) and Pauli repulsion [41]. The larger the ELF value, the more localized the electrons are, indicating that there is a covalent bond, the inner shells or a single pair of atoms whereas the lower values (< 0.5) describe areas where electrons should be delocalized [42].

**Fig. 3:**
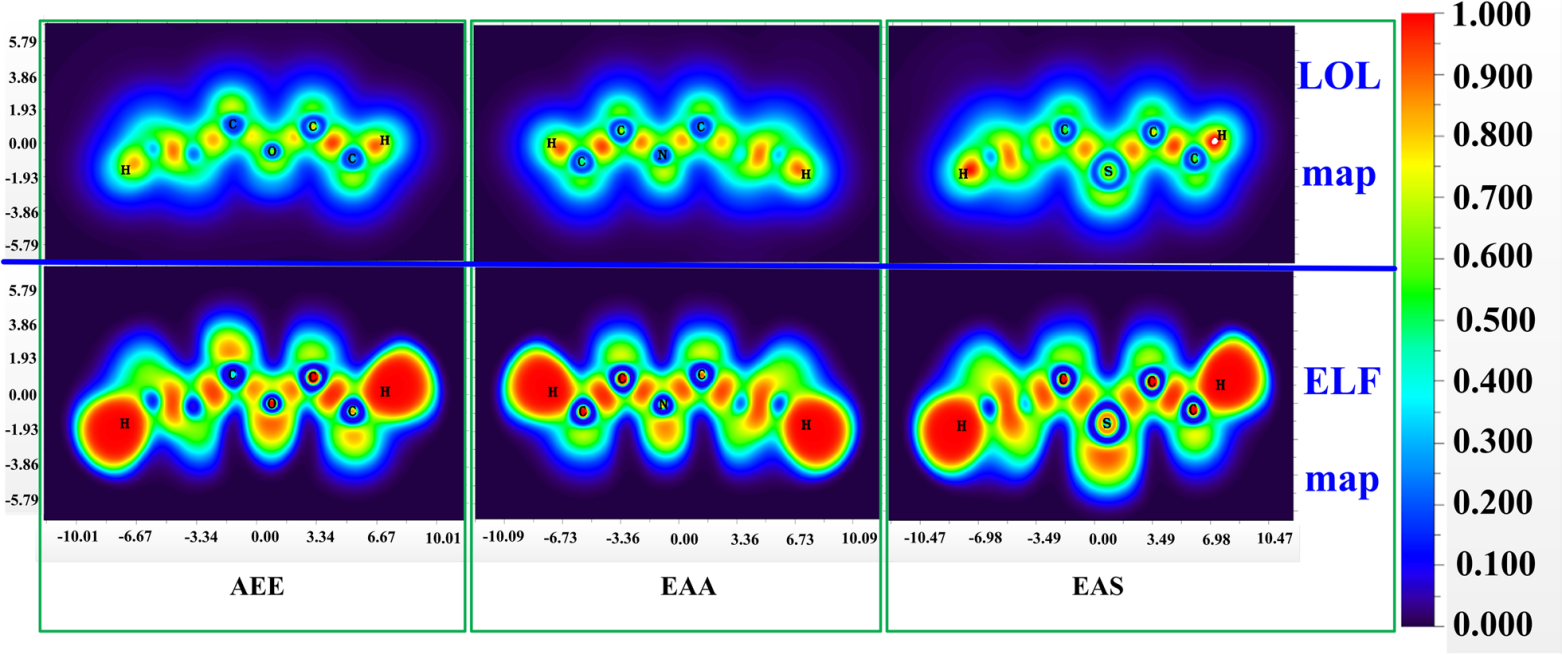
Visualization of localized orbital locator (LOL) part above the figure and below part depicted electron localization function (ELF) of the three title monomers: AEE, EAA and EAS

According to Fig. 3, the ELF color filled maps were shown in below part, shown from the top limit 1.0 value to the lower limit 0.0, represented by red and blue color, respectively. The red color areas near C–C, C–N and C–S bonds have higher density values that demonstrated the interaction with localized electron cloud; especially represented the hydrogen atoms in terminal carbon. The blue color region of few C atoms in AEE molecular reveals the delocalized bonding. In LOL part (Fig. 3 above part), large LOL values are depicted in red, it revealing covalent region between single bond such as C–C, C–S and C–N atoms. But the blue zone near the carbon atoms is describing the depletion of electrons between the valance and the inner shell.

### The transition of hydrogen abstraction reactions

In our early stage research, the hydrogen atom will migrate from the methylene carbon part in the allyl chain (–CH_2_–CH=CH_2_) to the benzoyl radical (BR). In order to investigate these hydrogen abstraction reactions in different solvents we used TST method, and we obtained the thermodynamic properties to describe the value of E_a_ and E_i_. For simplicity in the HAR process, we used the distortion/interaction model [43, 44] that includes the interacting energy (E_i_) and the deforming energy (E_d_) and represents the difference between them. Using the IRC calculation, we obtained the E_d_ energy which goes along with the structural deformation of the TS reaction complex [45]. We anatomized the components of the Δ_r_H, Δ_r_G, E_a_, E_d_ and E_i_ are as displayed in Table 1.

**Table 1:** The thermodynamic parameters in different hydrogen abstraction reactions enviroment, including ∆_r_*H*, ∆_r_*G, E*_a,_ *E*_d_ and *E*_i_ value

	∆_r_*H*	∆_r_*G*	*E*_a_	*E*_d_			*E*_i_
				Sum	Donor	Acceptor	
*AEE*							
Gas	− 10.38	− 10.35	20.20	13.13	12.58	0.55	7.06
Water	− 12.73	− 12.48	18.37	12.36	11.69	0.67	6.01
Methanol	− 12.73	− 12.39	18.63	12.29	11.63	0.66	6.33
DMSO	− 12.30	− 11.81	19.47	12.61	11.95	0.66	6.86
*EAA*							
Gas	− 13.12	− 13.00	18.96	14.88	14.34	0.54	4.07
Water	− 16.55	− 16.44	18.78	13.41	12.65	0.76	5.37
Methanol	− 17.30	− 17.09	19.55	13.22	12.50	0.72	6.34
DMSO	− 16.59	− 16.39	19.70	13.92	13.27	0.65	5.78
*EAS*							
Gas	− 9.05	− 9.03	18.63	15.33	14.83	0.50	3.30
Water	− 11.96	− 11.82	17.92	14.14	13.54	0.60	3.78
Methanol	− 11.80	− 11.59	18.55	14.18	13.58	0.60	4.38
DMSO	− 10.42	− 10.17	19.43	15.26	14.71	0.55	4.16

The definitions of these variables in Additional file 1

Δ_r_H, enthalpy change; △_r_G, Gibbs’ free energy change/reaction drive force; E_a_, activation energy; E_d_, deformation energy; E_i_, interaction energy E_a_ = E_d_ + E_i_ E_d_(sum) = E_d_(Donor) + E_d_(Acceptor) unit: kcal/mol

It is obviously seen in the Table 1 that the E_a_ values get a lower in the water solvent, such as a value with a 17.92 kcal/mol in the EAS and BR reaction group, especially. In the AEE systems, the E_a_ value of AEE + BR is the highest in gas phase, which reached 20.20 kcal/mol. However, the suspension polymerization of allyl ether in water solvent were found to perform better before our early study, and this holds very well in HAR. Compared the value of E_a_ in different solvents, it revealed that the higher value of E_a_ not only exist in gas phase. Moreover, it reveals that solvent effect in the water show more obvious.

On the other hand, the Δ_r_G (− 17.09 kcal/mol) in the EAA + BR group in methanol solvent is much more negative than other groups which having a large reaction driving force than others. Comparison of the Δ_r_G data indicated that in water and methanol groups values are more negative, revealing that the solvent effect in water and methanol have more reactivity due to a proton polar condition. The E_d_ values have been impacted not only by changes in connection angle, but also by changes in connection length [46]. Generally, a larger E_d_ value will result in a larger E_a_, that does not promote the appearance of the reaction. Compared to other solvents, the results reflected that the influence of gas phase increases the value of E_d_ of hydrogen abstraction reactions groups which also clarify the higher value of E_a_. Consequently, it can be expected that allyl monomers will be more active among HAR in a polar solvent.

Kinetic parameters, using Eckart’s correction formula, were derived from the calculation results in Table 2. The bond order (*n*_T_) is the "latency" or "earliness" criterion used by the TS. The higher *n*_T_ value, the more delayed the TS occurs. It was based on bond energy-bond order model (BEBO) [47] from the Eq. (1) below:

1 $$n_T = \frac{E_a }{{2E_a - \Delta H}}$$

**Table 2:** The kinetic parameters of the hydrogen abstraction reactions

	ω^≠^	*κ*(T)	*k* cm^3^∙molecule^−1^ s^−1^	*n*_T_
*AEE*				
Gas	− 1711.47	29.39	2.26 × 10^–20^	0.40
Water	− 1737.80	23.53	4.00 × 10^–19^	0.34
Methanol	− 1732.70	26.62	2.90 × 10^–19^	0.37
DMSO	− 1728.38	26.80	7.06 × 10^–20^	0.38
*EAA*				
Gas	− 1716.69	22.54	1.41 × 10^–19^	0.37
Water	− 1733.04	26.28	2.17 × 10^–19^	0.35
Methanol	− 1741.18	27.77	6.26 × 10^–20^	0.35
DMSO	− 1732.70	26.97	4.73 × 10^–20^	0.35
*EAS*				
Gas	− 1680.73	18.73	2.03 × 10^–19^	0.40
Water	− 1691.35	17.74	6.44 × 10^–19^	0.37
Methanol	− 1695.77	21.20	2.64 × 10^–19^	0.38
DMSO	− 1690.86	22.28	6.33 × 10^–20^	0.39

ω≠, imaginary frequency; κ(T), Eckart’s tunneling factor; k, rate constants; n_T_, bond order

The *κ*(T)is Eckart’s tunneling factor, is represented by the below Eq. (2).

2 $$\kappa (T) = 1 + \frac{1}{24}\left( {\frac{h\omega^\ne }{{k_{\text{b}} T}}} \right)^{2}$$

the imaginary frequency is *ω*^≠^; *T* is the temperature; *k*_b_ means Boltzmann constant and Planck constant is expressed by *h*.

With the aim of understanding reaction processes further we calculated the rate constants (*k*) value by TS optimization. The rate constants were expressed by the conventional TST formula Eq. (3) below:

3 $$k = \sigma \frac{{k_{\text{b}} T}}{h} \cdot \, \frac{q^\ne }{{q_A q_B }} \cdot \exp \left( { - \frac{E}{RT}} \right)$$

the symmetry factor σ = 2 owing to the two possible HAR route from donors by methylene group. As well as Eq. (2), the *q*^*≠*^, *q*_*A*_ and *q*_*B*_ are the partition functions, which represented the reactants and the transition state, A and B per unit volume. The calculated results of the kinetic parameters are displayed in different solvents and presented in Table 2.

According to the calculated value of n_T_, the appearance of TS of HAR in gas phase later than other solvents because of the greater value at 0.40. Peculiarly, the value *n*_*T*_ of AEE + BR group in water reaction is 0.34. It revealed that this group occurred to TS state is earlier than others. Besides, the *ω*^≠^ value of EAS + BR group is the lowest, therefore the corresponding κ(T) becomes quite low. We observed that the κ(T) value increases with the increase in Ea, and the *κ*(T) value in gas phase is greater. That means the lower E_a_ was easily obtained in solvents. Comparing the rate coefficients (*k*) in twelve reactions, such as EAS + BR group in water solvents reaction, the *k* value is 6.44 × 10^−19^ cm^3^ molecule^−1^ s^−1^ is the maximum. The kinetic parameters analysis investigation concluded that, the solvents effect is play a significant role in kinetic factor in HAR.

### The independent gradient model in Hirshfeld partition of molecular density analysis of complexes of transition state

There is a novel visual approach for investigating intra-fragment (δg^intra^) and the inter-fragment (δg^inter^) interactions between title compounds and BR in different solutions by IGMH study [48, 49]. Hence, the IGMH analysis is employed to obtain non-covalent complex interactions between AEE, EAA, EAS and BR in TS. For instance, the δg^inter^ gives a description of the interaction zones in TS. Diagram the function sign (I_2_) r (the result of the sign of the second maximum characteristic value of the electron density matrix in Hessen and the pro-molecular estimate with electron density) onto colored δg^inter^ isosurfaces of various colors. Inter-molecular interactions can be displayed in color by marking the real space function sign (I_2_) r, containing their strength and position. The Blue-Green–Red (BGR) represented the default color transition within a colored isosurface δg inter versus sign (I_2_) r. The bluer area is more conducive to interactions, like H–H bonds, while the greener zone is in lower attractive ability, like vdW interactions.

**Fig. 4:**
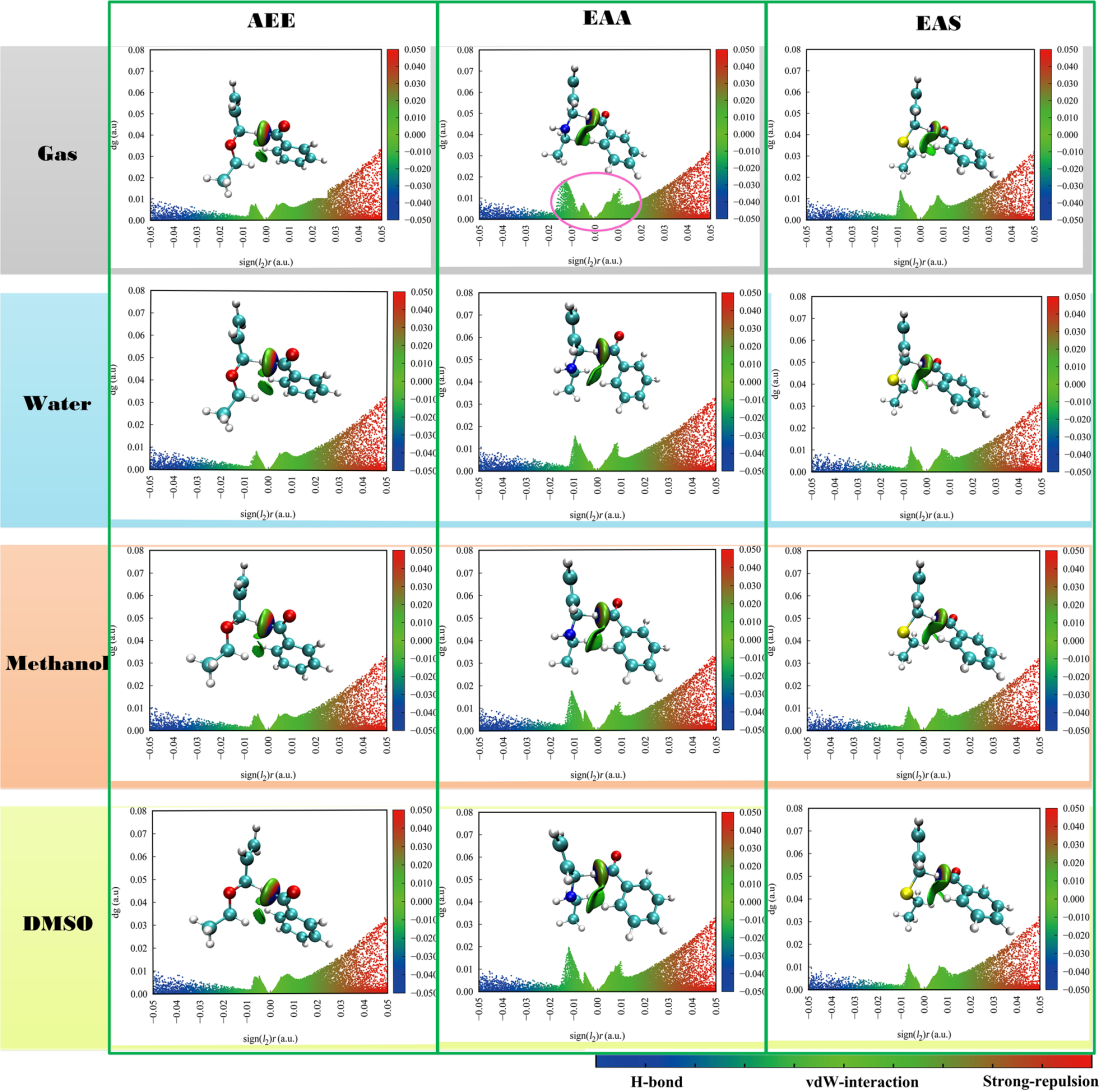
The visualized weak interaction areas (isosurface value = 0.005 a.u.) and the corresponding IGMH scatter map between donors and acceptors in different solvent systems

To graphically display the area of occurrence, the IGMH isosurfaces (isovalues = 0.005) was set. A good illustration of where the interaction is taking place between AEE, EAA, EAS and BR is provided by δg^inter^ in Fig. 4. On the IGMH isosurface map, the blue regions have the highest electrostatic attraction. According to the Fig. 4, The EAA IGMH isosurface was found to clearly display areas of low contact in green region (marked by pink circle part), and the elliptical slab derived from the N atoms of EAA and the H atom of BR molecular in gas phase, water, methanol and DMSO, as well as the S atom of EAS molecule obtained the less elliptical slab with the H atom of BR in different solvents. Compared to the scatter maps in different solvents, it could be found from Fig. 4 of sign(I_2_) r versus δg^inter^, sign (I_2_) r had reached more notable heights around − 0.013 a.u. in EAA molecular and BR part in four groups due to the larger attraction through vdW interaction zones represented green isosurfaces. The middle peaks relating to vdW interaction formation, the weak vdW interaction disappears in EAA and BR group in the water described in lower peak than other solvent systems. Compared with the H-bond of leftmost peak, there are almost no large peaks in all systems between AEE, EAA, EAS with BR molecular.

### Compare with rate constants of complexes reactions in solvents and temperature effect

**Fig. 5:**
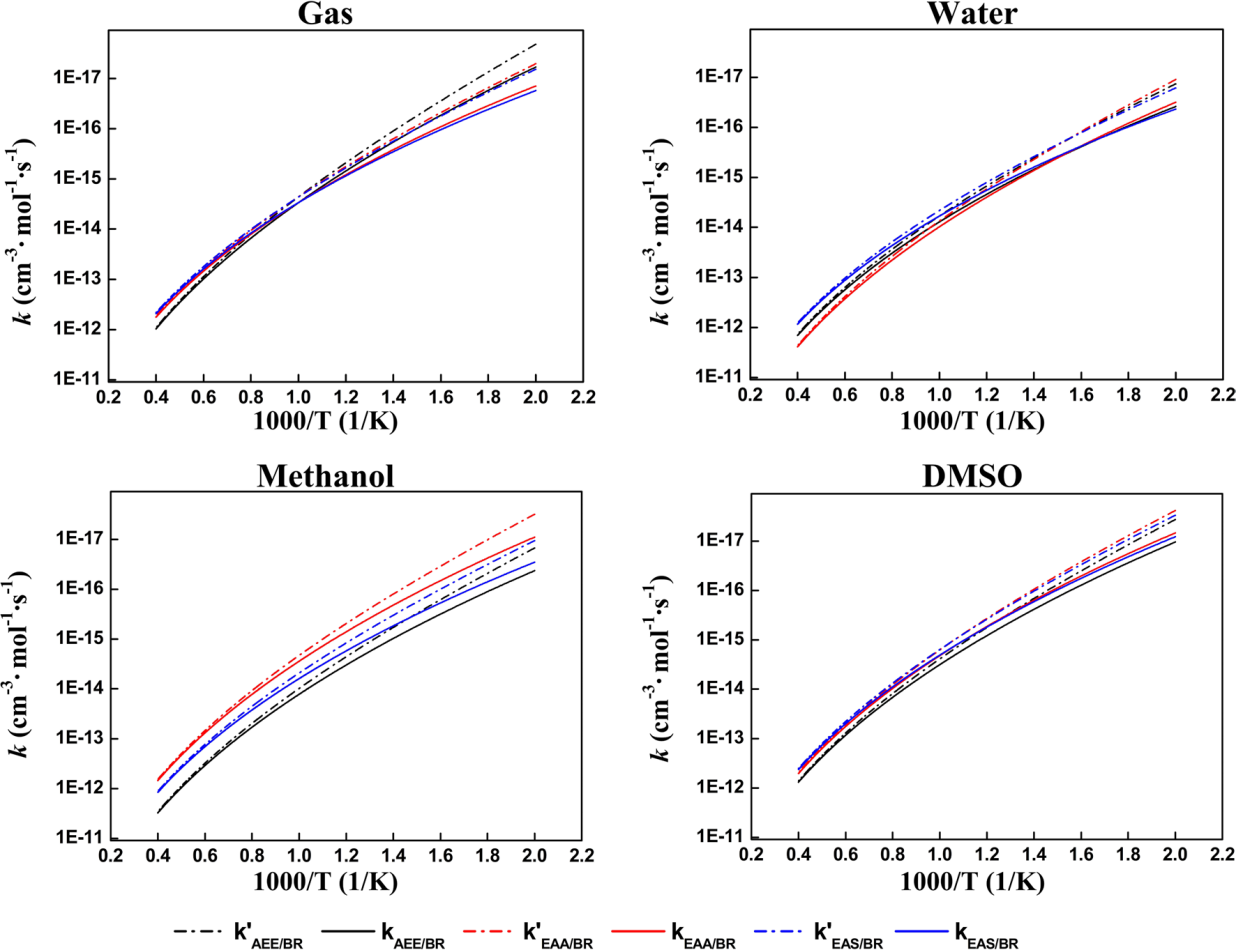
The rate constants parameters from the lower limit 500 K to the upper limit 2500 K. Dash line: without tunneling correction calculation data. Solid line: with Eckart’s method calculation data

As shown in Table 1, these reactions of BR + AEE/EAA/EAS systems have noticeably barriers. Each reaction is transferred from reagents to products through TSs and these groups’ rate coefficients are based on temperature are also obtained. According to Fig. 5, the rate constants for involved all reactions have a positive correlation with increasing temperature. Additional file 1: Table S4 shows the EAS group had a higher value (4.29 × 10^–17^) than the other in water solvent at 500 K, it maybe results from a lower barriers (Ea) in Table 1. While at the 1000 K, the EAS and BR reactions rate constant is the lowest than the other systems in water solvent.

In methanol solvent, it can be seen that these reactions rate constants value in solid line is comparatively closed to in the dash line in lower temperature and they also have a proportional growth with increasing temperature. In DMSO solvent, the EAA and EAS reactions with the same BR acceptors tend to have similarities in rate constants owing to slight difference in barrier heights. Besides, AEE + BR group in gas phase, the change of our calculation results for this reaction constant rates is 3 orders of magnitude of temperature variations range from 500 to 1000 K. Specially, this reaction rate line is sharper and it revealed that AEE reactions rate change has more sensitive when the temperature starts in gas phase. However, for EAA + BR group in water, methanol, and DMSO solvents, it has more sensitive with increasing temperature.

According to the Additional file 1: Table S4, at 1500 K, for group EAA + BR, our obtained rate constant value (1.43 × 10^–13^) of this reaction show one order magnitude of the other two reactions in water solvent. While at the 2000 K, the value of rate constant value for all reactions is of the same order of magnitude from Additional file 1: Table S4. Compare the rate constant value in methanol solvent reactions of group EAA + BR, its calculations results of rate constant is change more between 500 and 1000 K. For EAS + BR reactions, the rate constant is virtually the highest over the whole temperature range and these reactions are not more sensitive from 2000 to 2500 K. It revealed that the higher temperature is change more value for EAS + BR reaction in methanol solvent than other solvents. Besides, the change tendencies of reactions rate values are in conformity with temperature in different solvents.

## Conclusion

In our research before, HAR of multi-allyl ether monomers was speculated to be the first step in polymeric process. In this work, the title compounds were completely investigated by calculation data, the monomer AEE, EAA and EAS have different chemical properties. The allyl-type monomer AEE and EAS processed not only highly reactive ability but also stronger electron density than other systems. Besides, comparing the reactions of TSs in different solvents, we found the EAS system in water solvent have more chemical reactivity in HAR. Moreover, contrasting the rate constants, the AEE, EAA and EAS systems have a positive correlation with temperature. Especially, it shows an equative rate value of the increase in methanol solvents. Furthermore, our computational work is of paramount value and makes a fair contribution to the construction of the polymerizing process of multi-allyl-type monomer and search more excellent click monomers for HAR in the future.

### Supplementary Information

**Additional file 1**. Cartesian coordinates of the optimized structure (X, Y, Z). **Figure S1**. Chemical structures of the studied donors and acceptors. **Figure S2**. Schematic diagram of energy changes. **Figure S3**. IRC graphs of all the reactions in different solvents. **Figure S4**. The surface plots of vdW surface in green area from different solvents. **Table S1**. The values for highest occupied molecular orbital (HOMO) and lowest unoccupied molecular orbital (LUMO) of the AEE, EAA and EAS at the M06-2X/6-311++g(d,p) level. **Table S2**. The calculated quantum chemical parameters of title compounds. **Table S3**. Changes in bond angles and distances between the reaction complex (RC) and the transition state (TS). **Table S4**. The rate constants value with and without tunneling correction as a function of temperature from 500 K to 2500 K. **Table S5**. The calculated quantum chemical Hirshfeld charges and Fukui Function of title compounds. The definitions of abbreviation.

## Data Availability

The datasets used and/or analyzed during the current study available from the corresponding author on reasonable request.
