# Supplementary material for: DFT studies of solvent effect in hydrogen abstraction reactions from different allyl-type monomers with benzoyl radical
Source: BMC Chem. 2023 Sep 12;17(1):111. doi: 10.1186/s13065-023-01027-9 (PMC10496217; doi:10.1186/s13065-023-01027-9)
Supplement: Supplementary file 1 — Additional file 1. Cartesian coordinates of the optimized structure (X, Y, Z). Figure S1. Chemical structures of the studied donors and acceptors. Figure S2. Schematic diagram of energy changes. Figure S3. IRC graphs of all the reactions in different solvents. Figure S4. The surface plots of vdW surface in green area from different solvents. Table S1. The values for highest occupied molecular orbital (HOMO) and lowest unoccupied molecular orbital (LUMO) of the AEE, EAA and EAS at the M06-2X/6-311++g(d,p) level. Table S2. The calculated quantum chemical parameters of title compounds. Table S3. Changes in bond angles and distances between the reaction complex (RC) and the transition state (TS). Table S4. The rate constants value with and without tunneling correction as a function of temperature from 500 K to 2500 K. Table S5. The calculated quantum chemical Hirshfeld charges and Fukui Function of title compounds. The definitions of abbreviation. [file 13065_2023_1027_MOESM1_ESM.docx]

# *Supplementary Material*

# DFT studies of solvent effect in hydrogen abstraction reactions from different allyl-type monomers with Benzoyl radical

Xiaotian Zhao ^a†^, YaMing Li ^b†^, Shibo Lin ^a^, Chun Liu ^a^, Xirui Guo ^a^, Xuanhao Li ^a^, Lihui He ^a^, Xi Chen ^a^, Guodong Ye* ^c^

^a^ *Department of Pharmacy, Chengdu Second Peoples Hospital, Chengdu 610017, P.R. China*

^b^ *Department of Stomatology, Chengdu Second Peoples Hospital, Chengdu 610017, P.R. China*

^c^ *Guangzhou Municipal and Guangdong Provincial Key Laboratory of Molecular Target & Clinical Pharmacology, the NMPA and State Key Laboratory of Respiratory Disease, School of Pharmaceutical Sciences and the Fifth Affiliated Hospital, Guangzhou Medical University, Guangzhou 511436, China*

† *These authors have contributed equally to this work*

***Correspondence**:

Guodong Ye
Tel: +86-13229494539
email: [gzhygd@gzhmu.edu.cn](mailto:gzhygd@gzhmu.edu.cn)

***Keywords****:* allyl, solvent effect, DFT calculations, hydrogen abstraction reaction, transition state

### 1.Cartesian coordinates of the optimized structure (X,Y,Z)

#### AEE

| Molecular in gas phase |  |  |  |  |
| --- | --- | --- | --- | --- |
| \| H \|  \| -0.56577 \| 1.159729 \| 1.133726 \| \| --- \| --- \| --- \| --- \| --- \| \| C \|  \| -1.8797 \| -0.32271 \| 0.330882 \| \| H \|  \| -1.80126 \| -1.12761 \| 1.056649 \| \| C \|  \| -2.98905 \| -0.15501 \| -0.37669 \| \| H \|  \| -3.07338 \| 0.636584 \| -1.11464 \| \| H \|  \| -3.84962 \| -0.79924 \| -0.24337 \| \| C \|  \| -0.67535 \| 0.557944 \| 0.217683 \| \| H \|  \| -0.78681 \| 1.252069 \| -0.62731 \| \| C \|  \| 1.661806 \| 0.498031 \| 0.001244 \| \| H \|  \| 1.617192 \| 1.214299 \| -0.83166 \| \| H \|  \| 1.774122 \| 1.076198 \| 0.929878 \| \| C \|  \| 2.819896 \| -0.46035 \| -0.17832 \| \| H \|  \| 3.764292 \| 0.085559 \| -0.21965 \| \| H \|  \| 2.858593 \| -1.16469 \| 0.65383 \| \| H \|  \| 2.701196 \| -1.02575 \| -1.1036 \| \| O \|  \| 0.466976 \| -0.25183 \| 0.049666 \| |  |  |  |  |
|  |  |  |  |  |
| Molecular in water solvent |  |  |  |  |
| \| H \|  \| -0.547283 \| 1.245562 \| 1.062094 \| \| --- \| --- \| --- \| --- \| --- \| \| C \|  \| -1.89642 \| -0.267539 \| 0.381115 \| \| H \|  \| -1.872525 \| -0.954274 \| 1.224305 \| \| C \|  \| -2.964608 \| -0.202859 \| -0.40578 \| \| H \|  \| -2.998391 \| 0.476889 \| -1.252443 \| \| H \|  \| -3.839391 \| -0.817958 \| -0.225959 \| \| C \|  \| -0.681506 \| 0.584486 \| 0.194862 \| \| H \|  \| -0.781569 \| 1.209608 \| -0.700786 \| \| C \|  \| 1.664571 \| 0.489317 \| -0.020222 \| \| H \|  \| 1.603671 \| 1.162098 \| -0.884475 \| \| H \|  \| 1.777607 \| 1.105453 \| 0.880264 \| \| C \|  \| 2.820715 \| -0.473244 \| -0.166572 \| \| H \|  \| 3.758623 \| 0.08085 \| -0.237446 \| \| H \|  \| 2.878416 \| -1.139766 \| 0.696488 \| \| H \|  \| 2.707778 \| -1.07657 \| -1.069672 \| \| O \|  \| 0.457068 \| -0.259108 \| 0.075901 \| |  |  |  |  |
|  |  |  |  |  |
| Molecular in methanol solvent |  |  |  |  |
| \| H \|  \| 0.551792 \| 1.247047 \| -1.062792 \| \| --- \| --- \| --- \| --- \| --- \| \| C \|  \| 1.893177 \| -0.270982 \| -0.377563 \| \| H \|  \| 1.860296 \| -0.965798 \| -1.21436 \| \| C \|  \| 2.967128 \| -0.204187 \| 0.401185 \| \| H \|  \| 3.011559 \| 0.48235 \| 1.242324 \| \| H \|  \| 3.837887 \| -0.825495 \| 0.220486 \| \| C \|  \| 0.682035 \| 0.587224 \| -0.193295 \| \| H \|  \| 0.784207 \| 1.214863 \| 0.700853 \| \| C \|  \| -1.667079 \| 0.491341 \| 0.019184 \| \| H \|  \| -1.611561 \| 1.166527 \| 0.88258 \| \| H \|  \| -1.782853 \| 1.105263 \| -0.883112 \| \| C \|  \| -2.818025 \| -0.477709 \| 0.165971 \| \| H \|  \| -3.760339 \| 0.070071 \| 0.235041 \| \| H \|  \| -2.870047 \| -1.146593 \| -0.696199 \| \| H \|  \| -2.701617 \| -1.079327 \| 1.070317 \| \| O \|  \| -0.457843 \| -0.252879 \| -0.073504 \| |  |  |  |  |
|  |  |  |  |  |
| Molecular in DMSO solvent |  |  |  |  |
| \| H \|  \| -0.55314 \| 1.217701 \| 1.084544 \| \| --- \| --- \| --- \| --- \| --- \| \| C \|  \| -1.89007 \| -0.28455 \| 0.365938 \| \| H \|  \| -1.85026 \| -1.00901 \| 1.176673 \| \| C \|  \| -2.97374 \| -0.18628 \| -0.396 \| \| H \|  \| -3.02576 \| 0.529343 \| -1.21207 \| \| H \|  \| -3.84495 \| -0.80997 \| -0.2264 \| \| C \|  \| -0.67679 \| 0.574698 \| 0.200158 \| \| H \|  \| -0.78455 \| 1.225741 \| -0.67767 \| \| C \|  \| 1.66154 \| 0.489684 \| -0.00749 \| \| H \|  \| 1.611714 \| 1.187374 \| -0.85449 \| \| H \|  \| 1.776438 \| 1.085453 \| 0.90823 \| \| C \|  \| 2.820699 \| -0.46689 \| -0.17252 \| \| H \|  \| 3.759863 \| 0.088357 \| -0.22404 \| \| H \|  \| 2.87309 \| -1.15639 \| 0.672944 \| \| H \|  \| 2.712202 \| -1.04771 \| -1.09108 \| \| O \|  \| 0.45944 \| -0.25886 \| 0.062854 \| |  |  |  |  |

#### EAA

Molecular in gas phase

| H |  | 0.592301 | 1.351317 | -0.953201 |
| --- | --- | --- | --- | --- |
| C |  | 1.94952 | -0.199903 | -0.41464 |
| H |  | 1.935823 | -0.792156 | -1.326201 |
| C |  | 3.00204 | -0.237712 | 0.393938 |
| H |  | 3.026338 | 0.338798 | 1.313688 |
| H |  | 3.874169 | -0.838046 | 0.163994 |
| C |  | 0.715294 | 0.610817 | -0.153795 |
| H |  | 0.830556 | 1.170756 | 0.788904 |
| C |  | -1.703407 | 0.49144 | 0.071791 |
| H |  | -1.661603 | 1.078424 | 1.00446 |
| H |  | -1.82654 | 1.208529 | -0.746203 |
| C |  | -2.891667 | -0.456943 | 0.109406 |
| H |  | -3.823508 | 0.090854 | 0.260183 |
| H |  | -2.959029 | -1.015479 | -0.825429 |
| H |  | -2.789564 | -1.174515 | 0.928164 |
| N |  | -0.467037 | -0.241939 | -0.168536 |

Molecular in water solvent

| H |  | -0.589911 | 1.469145 | 0.772596 |
| --- | --- | --- | --- | --- |
| C |  | -1.969379 | -0.117198 | 0.440551 |
| H |  | -2.027512 | -0.522848 | 1.448661 |
| C |  | -2.980646 | -0.293101 | -0.404242 |
| H |  | -2.941401 | 0.098692 | -1.41695 |
| H |  | -3.878579 | -0.827046 | -0.112752 |
| C |  | -0.709917 | 0.616794 | 0.095223 |
| H |  | -0.786869 | 1.020417 | -0.924565 |
| C |  | 1.692593 | 0.471701 | -0.107756 |
| H |  | 1.597277 | 0.937417 | -1.099623 |
| H |  | 1.83507 | 1.283932 | 0.610538 |
| C |  | 2.894306 | -0.45521 | -0.093639 |
| H |  | 3.798813 | 0.085826 | -0.378681 |
| H |  | 3.048428 | -0.881809 | 0.900054 |
| H |  | 2.755575 | -1.276917 | -0.801823 |
| N |  | 0.466275 | -0.243147 | 0.258637 |

Molecular in methanol solvent

| H |  | -0.589756 | 1.361706 | 0.924935 |
| --- | --- | --- | --- | --- |
| C |  | -1.9587 | -0.187376 | 0.418598 |
| H |  | -1.97305 | -0.74632 | 1.352728 |
| C |  | -3.005789 | -0.233764 | -0.399102 |
| H |  | -3.011322 | 0.310895 | -1.33967 |
| H |  | -3.891468 | -0.811225 | -0.155341 |
| C |  | -0.712644 | 0.600185 | 0.145822 |
| H |  | -0.811066 | 1.129774 | -0.813839 |
| C |  | 1.700668 | 0.486237 | -0.067444 |
| H |  | 1.6359 | 1.056921 | -1.006772 |
| H |  | 1.822002 | 1.21567 | 0.739503 |
| C |  | 2.900821 | -0.44363 | -0.113552 |
| H |  | 3.817492 | 0.119043 | -0.30403 |
| H |  | 3.01447 | -0.977644 | 0.833061 |
| H |  | 2.787336 | -1.182687 | -0.91214 |
| N |  | 0.469004 | -0.262461 | 0.187809 |

Molecular in DMSO solvent

| H |  | 0.587747 | 1.352608 | -0.937228 |
| --- | --- | --- | --- | --- |
| C |  | 1.954577 | -0.195023 | -0.416687 |
| H |  | 1.956793 | -0.771017 | -1.340389 |
| C |  | 3.008398 | -0.232061 | 0.392728 |
| H |  | 3.024301 | 0.329404 | 1.323121 |
| H |  | 3.88893 | -0.818419 | 0.152079 |
| C |  | 0.713806 | 0.602807 | -0.147222 |
| H |  | 0.820095 | 1.145532 | 0.804393 |
| C |  | -1.703802 | 0.489815 | 0.063563 |
| H |  | -1.650155 | 1.073438 | 0.995711 |
| H |  | -1.820601 | 1.206732 | -0.755287 |
| C |  | -2.898292 | -0.447945 | 0.110602 |
| H |  | -3.821755 | 0.109645 | 0.28129 |
| H |  | -2.995038 | -0.997358 | -0.82896 |
| H |  | -2.787853 | -1.173663 | 0.921579 |
| N |  | -0.468516 | -0.257928 | -0.170999 |

#### EAS

Molecular in gas phase

| H |  | -0.69336 | 1.371772 | 0.912583 |
| --- | --- | --- | --- | --- |
| C |  | -2.22903 | -0.0366 | 0.437064 |
| H |  | -2.2744 | -0.52144 | 1.408757 |
| C |  | -3.26333 | -0.10961 | -0.39213 |
| H |  | -3.23377 | 0.354842 | -1.37263 |
| H |  | -4.17046 | -0.63481 | -0.11908 |
| C |  | -0.94963 | 0.669003 | 0.115004 |
| H |  | -1.0346 | 1.214676 | -0.82671 |
| C |  | 1.82047 | 0.602187 | 0.00659 |
| H |  | 1.711124 | 1.294947 | -0.83069 |
| H |  | 1.798268 | 1.17577 | 0.935883 |
| C |  | 3.121356 | -0.18125 | -0.11195 |
| H |  | 3.974448 | 0.499878 | -0.10606 |
| H |  | 3.234318 | -0.87684 | 0.721579 |
| H |  | 3.148081 | -0.75394 | -1.04063 |
| S |  | 0.408834 | -0.5492 | -0.00065 |

Molecular in water solvent

| H |  | -0.697209 | 1.340936 | 0.964361 |
| --- | --- | --- | --- | --- |
| C |  | -2.235852 | -0.046141 | 0.432631 |
| H |  | -2.304809 | -0.54555 | 1.396369 |
| C |  | -3.256685 | -0.095616 | -0.417646 |
| H |  | -3.206093 | 0.392918 | -1.386583 |
| H |  | -4.173026 | -0.619758 | -0.169777 |
| C |  | -0.951578 | 0.663729 | 0.145134 |
| H |  | -1.018872 | 1.232057 | -0.783863 |
| C |  | 1.82057 | 0.59956 | 0.001992 |
| H |  | 1.692866 | 1.289215 | -0.834395 |
| H |  | 1.797009 | 1.171981 | 0.931061 |
| C |  | 3.124548 | -0.172058 | -0.127473 |
| H |  | 3.966416 | 0.523341 | -0.129829 |
| H |  | 3.258603 | -0.863521 | 0.707205 |
| H |  | 3.154732 | -0.74388 | -1.05735 |
| S |  | 0.407773 | -0.554661 | 0.009685 |

Molecular in methanol solvent

| H |  | -0.696972 | 1.378029 | 0.92025 |
| --- | --- | --- | --- | --- |
| C |  | -2.236776 | -0.025176 | 0.436483 |
| H |  | -2.308344 | -0.480237 | 1.422171 |
| C |  | -3.252774 | -0.120375 | -0.41569 |
| H |  | -3.201096 | 0.321513 | -1.40713 |
| H |  | -4.167765 | -0.637937 | -0.147863 |
| C |  | -0.952876 | 0.675039 | 0.123104 |
| H |  | -1.022004 | 1.214399 | -0.823249 |
| C |  | 1.824139 | 0.597236 | -0.004056 |
| H |  | 1.699097 | 1.276752 | -0.849654 |
| H |  | 1.807724 | 1.182709 | 0.917465 |
| C |  | 3.123911 | -0.183091 | -0.126868 |
| H |  | 3.969905 | 0.508008 | -0.13801 |
| H |  | 3.25649 | -0.866343 | 0.715365 |
| H |  | 3.150368 | -0.765463 | -1.050784 |
| S |  | 0.404928 | -0.549577 | 0.022725 |

Molecular in DMSO solvent

| H |  | -0.697311 | 1.363604 | 0.937836 |
| --- | --- | --- | --- | --- |
| C |  | -2.236505 | -0.033537 | 0.435098 |
| H |  | -2.306918 | -0.506326 | 1.412365 |
| C |  | -3.254521 | -0.110664 | -0.41646 |
| H |  | -3.203638 | 0.349803 | -1.399342 |
| H |  | -4.170054 | -0.630971 | -0.156352 |
| C |  | -0.952528 | 0.670645 | 0.131838 |
| H |  | -1.021021 | 1.221646 | -0.807728 |
| C |  | 1.82296 | 0.598119 | -0.001693 |
| H |  | 1.697132 | 1.28176 | -0.84369 |
| H |  | 1.803796 | 1.1785 | 0.922866 |
| C |  | 3.124266 | -0.178851 | -0.127006 |
| H |  | 3.9687 | 0.51399 | -0.134845 |
| H |  | 3.257591 | -0.865212 | 0.712429 |
| H |  | 3.152361 | -0.757282 | -1.05323 |
| S |  | 0.406083 | -0.551487 | 0.017439 |

#### AEE+BR group

TS in Hydrogen Abstraction Reaction in gas phase

| H | X  0.928688 | Y  -0.617221 | Z  -0.054371 |
| --- | --- | --- | --- |
| C | 2.945387 | -1.503174 | -0.227776 |
| H | 3.346379 | -1.540156 | 0.78122 |
| C | 3.119931 | -2.525501 | -1.068462 |
| H | 2.724945 | -2.500876 | -2.079118 |
| H | 3.659882 | -3.414693 | -0.769768 |
| C | 2.140749 | -0.321088 | -0.535132 |
| H | 1.968934 | -0.139526 | -1.604418 |
| C | -1.530572 | -0.4438 | 0.239926 |
| C | -2.686283 | -0.631746 | 1.002238 |
| C | -1.596437 | 0.188009 | -0.999774 |
| C | -3.905813 | -0.183279 | 0.517295 |
| H | -2.605814 | -1.12766 | 1.962798 |
| C | -2.8208 | 0.635523 | -1.483783 |
| H | -0.6885 | 0.322576 | -1.578509 |
| C | -3.971521 | 0.449708 | -0.724088 |
| H | -4.807629 | -0.324077 | 1.101068 |
| H | -2.879147 | 1.125892 | -2.4478 |
| H | -4.926357 | 0.798682 | -1.099616 |
| C | -0.213897 | -0.918387 | 0.748411 |
| O | -0.019468 | -1.483231 | 1.775714 |
| C | 1.732124 | 1.938297 | -0.065758 |
| H | 1.685668 | 2.15002 | -1.143641 |
| H | 0.71057 | 1.726393 | 0.278156 |
| C | 2.322637 | 3.108599 | 0.689932 |
| H | 1.711815 | 4.001233 | 0.543828 |
| H | 2.365272 | 2.884147 | 1.756424 |
| H | 3.334447 | 3.314086 | 0.33806 |
| O | 2.551694 | 0.806007 | 0.156476 |
|  |  |  |  |

TS in Hydrogen Abstraction Reaction in water solvent

| H | 0.935761 | -0.610684 | -0.06782 |
| --- | --- | --- | --- |
| C | 2.946805 | -1.482628 | -0.258481 |
| H | 3.378848 | -1.546424 | 0.737858 |
| C | 3.102511 | -2.486194 | -1.127717 |
| H | 2.677496 | -2.438309 | -2.125934 |
| H | 3.655717 | -3.379265 | -0.862235 |
| C | 2.129562 | -0.29806 | -0.524435 |
| H | 1.943794 | -0.0773 | -1.580889 |
| C | -1.521737 | -0.474747 | 0.22044 |
| C | -2.696846 | -0.753205 | 0.928034 |
| C | -1.553856 | 0.302578 | -0.938156 |
| C | -3.903681 | -0.247192 | 0.468626 |
| H | -2.651109 | -1.359119 | 1.825905 |
| C | -2.767234 | 0.805684 | -1.394398 |
| H | -0.631197 | 0.507128 | -1.471506 |
| C | -3.936777 | 0.530731 | -0.690469 |
| H | -4.820085 | -0.455001 | 1.00808 |
| H | -2.80199 | 1.409485 | -2.292963 |
| H | -4.882601 | 0.9237 | -1.045186 |
| C | -0.222853 | -0.999136 | 0.686685 |
| O | -0.022547 | -1.690059 | 1.6447 |
| C | 1.68901 | 1.948998 | 0.030216 |
| H | 1.58082 | 2.151135 | -1.04183 |
| H | 0.698 | 1.712206 | 0.43511 |
| C | 2.300712 | 3.128569 | 0.748765 |
| H | 1.657892 | 4.003271 | 0.634292 |
| H | 2.408387 | 2.916812 | 1.81443 |
| H | 3.283011 | 3.36491 | 0.334914 |
| O | 2.544242 | 0.818193 | 0.205688 |

TS in Hydrogen Abstraction Reaction in methanol solvent

| H | 0.925677 | -0.597271 | -0.112518 |
| --- | --- | --- | --- |
| C | 2.917084 | -1.523273 | -0.227474 |
| H | 3.319568 | -1.571015 | 0.78252 |
| C | 3.072454 | -2.55266 | -1.065839 |
| H | 2.676549 | -2.522871 | -2.077079 |
| H | 3.597403 | -3.451051 | -0.761956 |
| C | 2.138176 | -0.324677 | -0.544117 |
| H | 1.995144 | -0.119633 | -1.610684 |
| C | -1.533865 | -0.43879 | 0.230068 |
| C | -2.684565 | -0.664685 | 0.994231 |
| C | -1.607651 | 0.239222 | -0.987162 |
| C | -3.909151 | -0.206356 | 0.531571 |
| H | -2.60586 | -1.193967 | 1.937566 |
| C | -2.838428 | 0.69598 | -1.445928 |
| H | -0.703185 | 0.402828 | -1.564382 |
| C | -3.983913 | 0.47278 | -0.686282 |
| H | -4.807515 | -0.374838 | 1.114181 |
| H | -2.90574 | 1.223214 | -2.390166 |
| H | -4.9437 | 0.829293 | -1.043311 |
| C | -0.216895 | -0.915535 | 0.698548 |
| O | 0.01787 | -1.511123 | 1.709789 |
| C | 1.76089 | 1.950138 | -0.062492 |
| H | 1.743862 | 2.15163 | -1.140406 |
| H | 0.732245 | 1.755905 | 0.265812 |
| C | 2.363228 | 3.107488 | 0.69912 |
| H | 1.770127 | 4.008991 | 0.531802 |
| H | 2.377401 | 2.897224 | 1.770833 |
| H | 3.385014 | 3.298636 | 0.363813 |
| O | 2.554483 | 0.789265 | 0.181274 |

TS in Hydrogen Abstraction Reaction in DMSO solvent

| H | 0.929319 | -0.617565 | -0.017988 |
| --- | --- | --- | --- |
| C | 2.915661 | -1.538107 | -0.277633 |
| H | 3.415876 | -1.574057 | 0.687941 |
| C | 2.981692 | -2.583028 | -1.109529 |
| H | 2.487836 | -2.566889 | -2.077057 |
| H | 3.529052 | -3.480097 | -0.843933 |
| C | 2.113821 | -0.340694 | -0.533043 |
| H | 1.883196 | -0.14814 | -1.587548 |
| C | -1.525445 | -0.42419 | 0.264836 |
| C | -2.706479 | -0.643974 | 0.982324 |
| C | -1.551478 | 0.248631 | -0.95696 |
| C | -3.911765 | -0.186032 | 0.470042 |
| H | -2.667433 | -1.169475 | 1.930023 |
| C | -2.762413 | 0.707033 | -1.465301 |
| H | -0.62584 | 0.409948 | -1.499947 |
| C | -3.93798 | 0.488941 | -0.751765 |
| H | -4.832472 | -0.351533 | 1.01748 |
| H | -2.790429 | 1.231488 | -2.413009 |
| H | -4.882091 | 0.845895 | -1.147621 |
| C | -0.225392 | -0.904779 | 0.790816 |
| O | -0.034591 | -1.471892 | 1.822851 |
| C | 1.747872 | 1.917691 | -0.027143 |
| H | 1.626955 | 2.118579 | -1.099413 |
| H | 0.754298 | 1.709951 | 0.390972 |
| C | 2.390835 | 3.091635 | 0.673644 |
| H | 1.766566 | 3.980081 | 0.557487 |
| H | 2.505511 | 2.887151 | 1.740246 |
| H | 3.374563 | 3.302115 | 0.248688 |
| O | 2.578532 | 0.774866 | 0.146143 |

#### EAA+BR group

TS in Hydrogen Abstraction Reaction in gas phase

| H | -1.035689 | -0.799252 | 0.385941 |
| --- | --- | --- | --- |
| C | -2.618652 | -1.158003 | -1.148607 |
| H | -2.225998 | -0.795731 | -2.097081 |
| C | -3.322393 | -2.293906 | -1.1134 |
| H | -3.722489 | -2.679671 | -0.181528 |
| H | -3.510214 | -2.869233 | -2.011052 |
| C | -2.254984 | -0.367858 | 0.027508 |
| H | -2.813744 | -0.625811 | 0.938106 |
| C | 1.396038 | -0.600166 | 0.250291 |
| C | 2.68649 | -1.000956 | 0.608026 |
| C | 1.196427 | 0.413601 | -0.686425 |
| C | 3.779966 | -0.382207 | 0.021706 |
| H | 2.808007 | -1.79179 | 1.339421 |
| C | 2.299222 | 1.032288 | -1.267427 |
| H | 0.183464 | 0.714286 | -0.942563 |
| C | 3.584527 | 0.633864 | -0.914695 |
| H | 4.785002 | -0.685633 | 0.289663 |
| H | 2.156599 | 1.822412 | -1.994808 |
| H | 4.441967 | 1.115596 | -1.370332 |
| C | 0.214113 | -1.254771 | 0.870144 |
| O | 0.227897 | -2.086894 | 1.719569 |
| C | -1.735296 | 1.807378 | 0.970568 |
| H | -2.50452 | 1.707071 | 1.751856 |
| H | -0.809986 | 1.392583 | 1.385699 |
| C | -1.527955 | 3.271187 | 0.616646 |
| H | -1.268731 | 3.851985 | 1.503123 |
| H | -0.724662 | 3.374267 | -0.115513 |
| H | -2.437811 | 3.700836 | 0.188607 |
| N | -2.067039 | 1.031968 | -0.217737 |
| H | -2.86012 | 1.436741 | -0.70794 |

TS in Hydrogen Abstraction Reaction in water solvent

| H | -1.080423 | -0.710135 | 0.178285 |
| --- | --- | --- | --- |
| C | -2.945298 | -1.016749 | -0.953418 |
| H | -2.783616 | -0.700203 | -1.982211 |
| C | -3.650552 | -2.126542 | -0.706841 |
| H | -3.828644 | -2.466163 | 0.309378 |
| H | -4.065662 | -2.72127 | -1.512236 |
| C | -2.292517 | -0.212922 | 0.08234 |
| H | -2.654688 | -0.422777 | 1.098244 |
| C | 1.406246 | -0.711495 | 0.110894 |
| C | 2.643885 | -1.269148 | 0.452033 |
| C | 1.335667 | 0.495878 | -0.585983 |
| C | 3.80991 | -0.609213 | 0.093309 |
| H | 2.6783 | -2.207919 | 0.993245 |
| C | 2.508587 | 1.152852 | -0.942051 |
| H | 0.364271 | 0.908399 | -0.840482 |
| C | 3.740415 | 0.600114 | -0.601553 |
| H | 4.773934 | -1.030557 | 0.352374 |
| H | 2.463419 | 2.090759 | -1.482297 |
| H | 4.654718 | 1.112623 | -0.877952 |
| C | 0.148245 | -1.389829 | 0.485988 |
| O | 0.041992 | -2.429165 | 1.074366 |
| C | -1.567841 | 1.930835 | 0.903904 |
| H | -2.264431 | 1.912658 | 1.75408 |
| H | -0.654195 | 1.427801 | 1.236594 |
| C | -1.258261 | 3.363421 | 0.510741 |
| H | -0.89288 | 3.926734 | 1.371619 |
| H | -0.495552 | 3.394373 | -0.271441 |
| H | -2.155578 | 3.863112 | 0.135393 |
| N | -2.099125 | 1.174063 | -0.23 |
| H | -2.97195 | 1.594228 | -0.5437 |

TS in Hydrogen Abstraction Reaction in methanol solvent

| H | -1.0428 | -0.762144 | 0.288177 |
| --- | --- | --- | --- |
| C | -2.716773 | -1.139069 | -1.099661 |
| H | -2.426961 | -0.779075 | -2.085885 |
| C | -3.386985 | -2.291127 | -0.985932 |
| H | -3.690252 | -2.674415 | -0.015557 |
| H | -3.649202 | -2.879168 | -1.858356 |
| C | -2.26162 | -0.332522 | 0.036058 |
| H | -2.743442 | -0.590283 | 0.990106 |
| C | 1.421637 | -0.629073 | 0.20525 |
| C | 2.698709 | -1.06655 | 0.575232 |
| C | 1.259468 | 0.448455 | -0.667235 |
| C | 3.813572 | -0.418336 | 0.064864 |
| H | 2.802258 | -1.905856 | 1.254366 |
| C | 2.382327 | 1.095181 | -1.173115 |
| H | 0.257234 | 0.770994 | -0.935982 |
| C | 3.653625 | 0.660818 | -0.807141 |
| H | 4.808274 | -0.747495 | 0.342684 |
| H | 2.267934 | 1.933561 | -1.850284 |
| H | 4.528829 | 1.164136 | -1.202852 |
| C | 0.214856 | -1.30057 | 0.729672 |
| O | 0.179308 | -2.209836 | 1.508268 |
| C | -1.715823 | 1.837612 | 0.950383 |
| H | -2.444541 | 1.723307 | 1.766835 |
| H | -0.767559 | 1.424818 | 1.312702 |
| C | -1.544194 | 3.306139 | 0.604254 |
| H | -1.258987 | 3.878952 | 1.489274 |
| H | -0.770065 | 3.437035 | -0.156342 |
| H | -2.478491 | 3.724182 | 0.218199 |
| N | -2.111082 | 1.068384 | -0.226869 |
| H | -2.961919 | 1.455701 | -0.630916 |

TS in Hydrogen Abstraction Reaction in DMSO solvent

| H | -1.039718 | -0.771261 | 0.325942 |
| --- | --- | --- | --- |
| C | -2.677522 | -1.141785 | -1.116434 |
| H | -2.35647 | -0.774004 | -2.090157 |
| C | -3.347445 | -2.297276 | -1.033084 |
| H | -3.682562 | -2.687361 | -0.076038 |
| H | -3.578013 | -2.880414 | -1.917494 |
| C | -2.261194 | -0.340023 | 0.03572 |
| H | -2.772103 | -0.599007 | 0.974052 |
| C | 1.411325 | -0.623151 | 0.222984 |
| C | 2.695318 | -1.050139 | 0.579467 |
| C | 1.233374 | 0.442413 | -0.660853 |
| C | 3.800967 | -0.404829 | 0.044993 |
| H | 2.813027 | -1.879776 | 1.26776 |
| C | 2.34694 | 1.086396 | -1.191311 |
| H | 0.226533 | 0.759595 | -0.919535 |
| C | 3.625245 | 0.66206 | -0.838626 |
| H | 4.800456 | -0.727146 | 0.31323 |
| H | 2.219349 | 1.915412 | -1.877522 |
| H | 4.492673 | 1.163155 | -1.25346 |
| C | 0.211959 | -1.294726 | 0.775895 |
| O | 0.188398 | -2.192307 | 1.561905 |
| C | -1.724933 | 1.831812 | 0.960751 |
| H | -2.474272 | 1.729689 | 1.759936 |
| H | -0.788776 | 1.413423 | 1.347611 |
| C | -1.531531 | 3.295667 | 0.605283 |
| H | -1.260251 | 3.875312 | 1.490064 |
| H | -0.739843 | 3.410079 | -0.139684 |
| H | -2.452858 | 3.718062 | 0.193918 |
| N | -2.097029 | 1.059977 | -0.221235 |
| H | -2.930173 | 1.454335 | -0.65392 |

#### EAS+BR group

TS in Hydrogen Abstraction Reaction in gas phase

| H | 0.742171 | 0.803205 | 0.537813 |
| --- | --- | --- | --- |
| C | 1.912303 | 1.966899 | -0.97239 |
| H | 1.531819 | 1.687337 | -1.952715 |
| C | 2.222953 | 3.24334 | -0.727955 |
| H | 2.607397 | 3.555667 | 0.237234 |
| H | 2.100212 | 4.00644 | -1.486135 |
| C | 1.976348 | 0.899085 | 0.029719 |
| H | 2.53681 | 1.1597 | 0.930142 |
| C | -1.633419 | 0.312083 | 0.40487 |
| C | -2.937974 | 0.383319 | 0.901761 |
| C | -1.375256 | -0.253683 | -0.84288 |
| C | -3.984761 | -0.114224 | 0.140947 |
| H | -3.106923 | 0.829137 | 1.87524 |
| C | -2.42945 | -0.755658 | -1.598809 |
| H | -0.354314 | -0.307704 | -1.209748 |
| C | -3.728757 | -0.683378 | -1.107224 |
| H | -5.000478 | -0.062629 | 0.514376 |
| H | -2.238328 | -1.20023 | -2.567798 |
| H | -4.549896 | -1.072003 | -1.698294 |
| C | -0.500112 | 0.842047 | 1.205183 |
| O | -0.548901 | 1.289325 | 2.304978 |
| C | 1.8465 | -1.748336 | 0.773254 |
| H | 2.306357 | -1.363456 | 1.685767 |
| H | 0.761587 | -1.657976 | 0.869474 |
| C | 2.248084 | -3.195956 | 0.527077 |
| H | 1.912198 | -3.824522 | 1.353734 |
| H | 1.798014 | -3.575277 | -0.392434 |
| H | 3.331815 | -3.291173 | 0.441785 |
| S | 2.394626 | -0.710897 | -0.623724 |

TS in Hydrogen Abstraction Reaction in water solvent

| H | 0.750708 | 0.741611 | 0.581434 |
| --- | --- | --- | --- |
| C | 1.923634 | 2.053939 | -0.788343 |
| H | 1.497704 | 1.89707 | -1.77763 |
| C | 2.312223 | 3.280531 | -0.423435 |
| H | 2.743779 | 3.46676 | 0.555426 |
| H | 2.207179 | 4.125132 | -1.09461 |
| C | 1.970402 | 0.886993 | 0.099258 |
| H | 2.531884 | 1.04472 | 1.022801 |
| C | -1.634248 | 0.261376 | 0.446228 |
| C | -2.909903 | 0.133069 | 1.00818 |
| C | -1.428952 | 0.047517 | -0.917749 |
| C | -3.978841 | -0.210751 | 0.194705 |
| H | -3.047902 | 0.30436 | 2.06974 |
| C | -2.505128 | -0.300338 | -1.726758 |
| H | -0.432693 | 0.150348 | -1.33621 |
| C | -3.774732 | -0.427487 | -1.16972 |
| H | -4.971603 | -0.312176 | 0.616581 |
| H | -2.355782 | -0.470557 | -2.785974 |
| H | -4.613932 | -0.69688 | -1.8006 |
| C | -0.477986 | 0.625872 | 1.287268 |
| O | -0.469031 | 0.814536 | 2.468978 |
| C | 1.803069 | -1.823108 | 0.573469 |
| H | 2.207659 | -1.49232 | 1.532001 |
| H | 0.712478 | -1.773588 | 0.618544 |
| C | 2.265058 | -3.232024 | 0.238629 |
| H | 1.898981 | -3.928562 | 0.995428 |
| H | 1.879704 | -3.554242 | -0.731285 |
| H | 3.354808 | -3.29398 | 0.21554 |
| S | 2.375107 | -0.656095 | -0.710213 |
|  |  |  |  |

TS in Hydrogen Abstraction Reaction in methanol solvent

| H | 0.730353 | 0.724947 | 0.576876 |
| --- | --- | --- | --- |
| C | 1.855574 | 2.09873 | -0.77365 |
| H | 1.455101 | 1.934685 | -1.772695 |
| C | 2.174204 | 3.339009 | -0.387749 |
| H | 2.578841 | 3.536691 | 0.600732 |
| H | 2.037399 | 4.185435 | -1.051556 |
| C | 1.944964 | 0.924227 | 0.101108 |
| H | 2.496309 | 1.093924 | 1.02907 |
| C | -1.653441 | 0.211768 | 0.448838 |
| C | -2.924321 | 0.07047 | 1.018615 |
| C | -1.457764 | 0.017067 | -0.919285 |
| C | -3.998005 | -0.26697 | 0.208658 |
| H | -3.054944 | 0.227392 | 2.083737 |
| C | -2.538542 | -0.324902 | -1.724854 |
| H | -0.46485 | 0.129969 | -1.34361 |
| C | -3.803375 | -0.46473 | -1.160094 |
| H | -4.987512 | -0.378075 | 0.636818 |
| H | -2.39634 | -0.480635 | -2.787705 |
| H | -4.646577 | -0.729247 | -1.788434 |
| C | -0.492918 | 0.570982 | 1.287258 |
| O | -0.477203 | 0.737498 | 2.47119 |
| C | 1.919489 | -1.793288 | 0.561566 |
| H | 2.341829 | -1.462728 | 1.512904 |
| H | 0.829613 | -1.780222 | 0.640496 |
| C | 2.415895 | -3.182869 | 0.195278 |
| H | 2.097938 | -3.898978 | 0.956254 |
| H | 2.009263 | -3.508601 | -0.765177 |
| H | 3.506164 | -3.20991 | 0.136101 |
| S | 2.414655 | -0.5926 | -0.722967 |
|  |  |  |  |

TS in Hydrogen Abstraction Reaction in DMSO solvent

| H | 0.731971 | 0.727864 | 0.588949 |
| --- | --- | --- | --- |
| C | 1.861015 | 2.082006 | -0.788693 |
| H | 1.456951 | 1.90691 | -1.784383 |
| C | 2.177188 | 3.3278 | -0.416479 |
| H | 2.585779 | 3.536215 | 0.568105 |
| H | 2.034459 | 4.167071 | -1.087868 |
| C | 1.954673 | 0.91761 | 0.096342 |
| H | 2.510311 | 1.095223 | 1.020092 |
| C | -1.63881 | 0.224922 | 0.451088 |
| C | -2.915245 | 0.096022 | 1.010052 |
| C | -1.432481 | 0.010598 | -0.912231 |
| C | -3.983498 | -0.248258 | 0.195192 |
| H | -3.055913 | 0.267045 | 2.071486 |
| C | -2.507429 | -0.337892 | -1.723171 |
| H | -0.435704 | 0.113147 | -1.329923 |
| C | -3.778096 | -0.465228 | -1.168796 |
| H | -4.976949 | -0.349738 | 0.616341 |
| H | -2.355842 | -0.508465 | -2.782369 |
| H | -4.616503 | -0.734898 | -1.801105 |
| C | -0.481555 | 0.592179 | 1.297557 |
| O | -0.473154 | 0.778424 | 2.474633 |
| C | 1.861681 | -1.799174 | 0.562479 |
| H | 2.25375 | -1.465055 | 1.525471 |
| H | 0.769851 | -1.776519 | 0.603035 |
| C | 2.358951 | -3.195069 | 0.223418 |
| H | 2.006913 | -3.903729 | 0.976193 |
| H | 1.985242 | -3.523298 | -0.749401 |
| H | 3.450299 | -3.231276 | 0.204524 |
| S | 2.411391 | -0.611311 | -0.712298 |

### 2. Figures and Tables

**
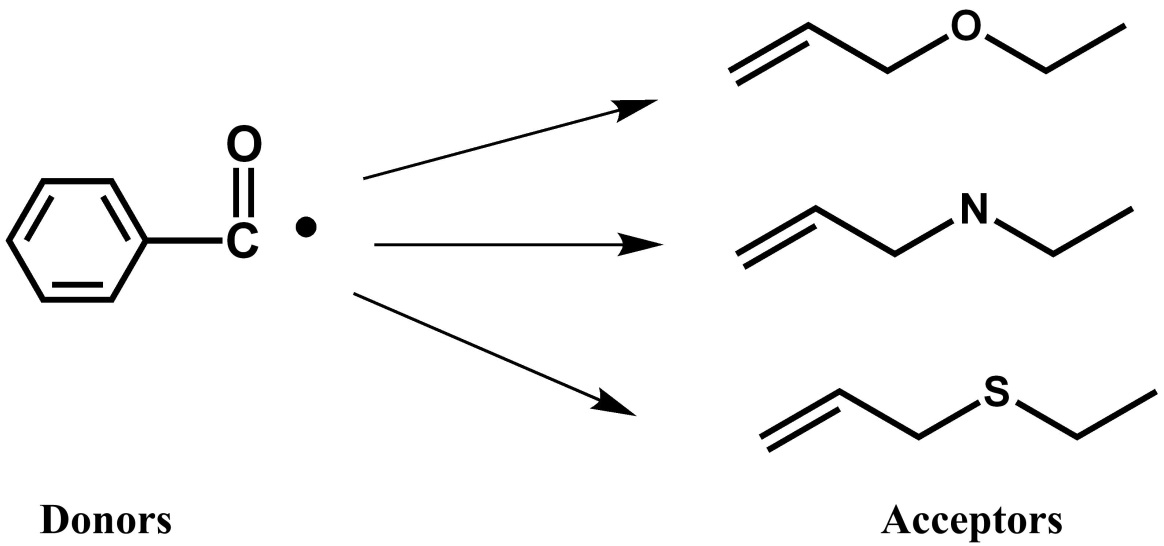
**

**Figure S1** Chemical structures of the studied donors and acceptors

**
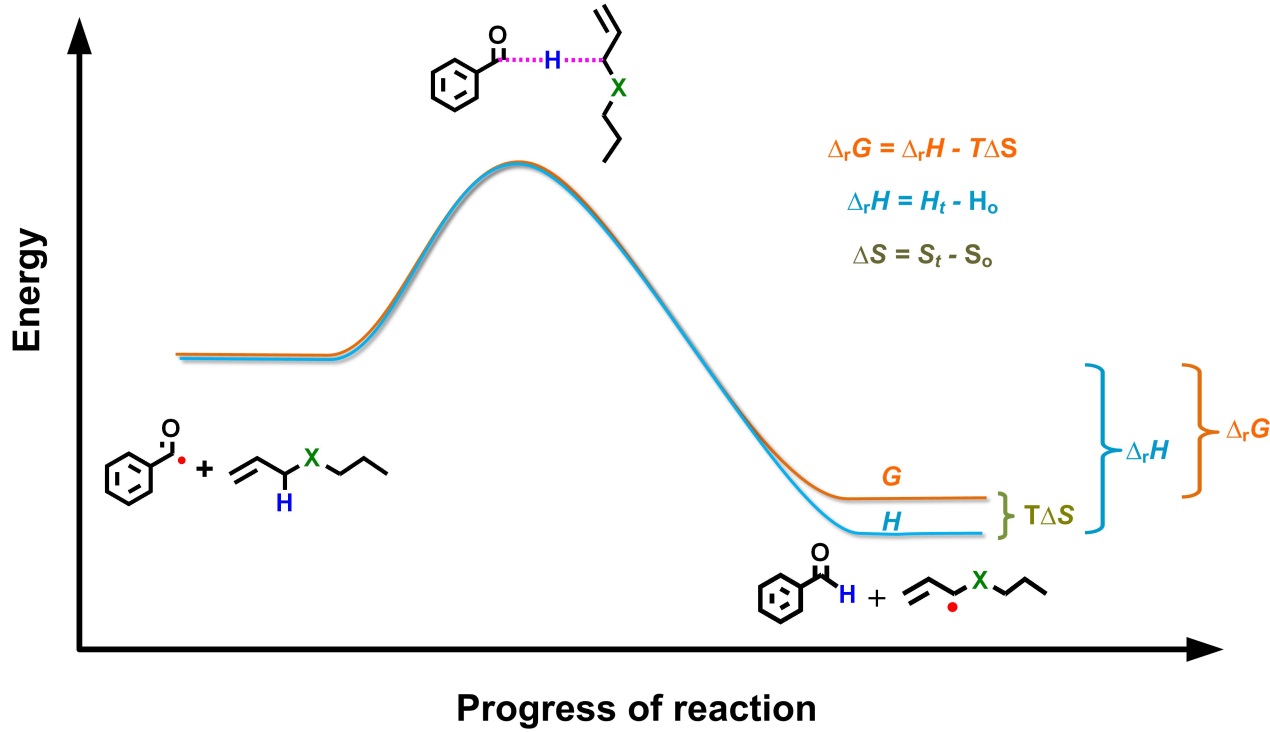
**

**Figure S2** Schematic diagram of energy changes

**
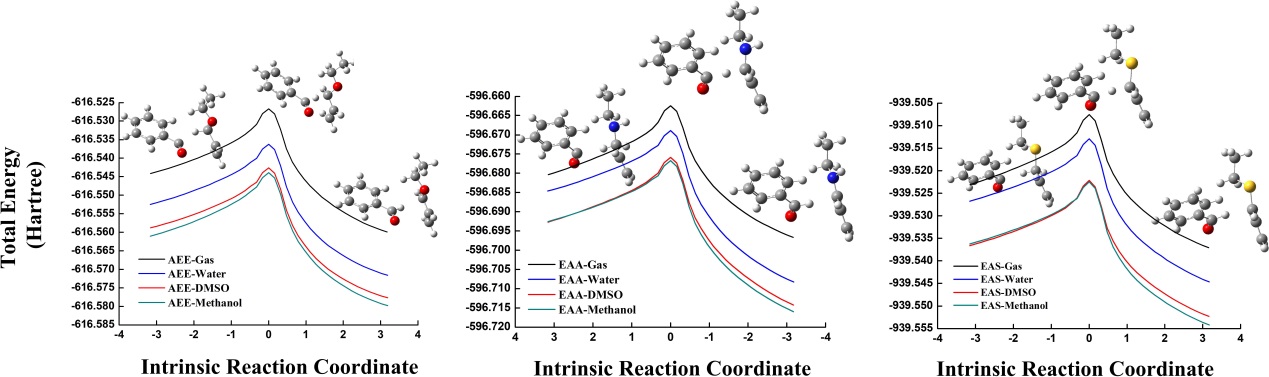
**

**Figure S3** IRC graphs of all the reactions in different solvents

**
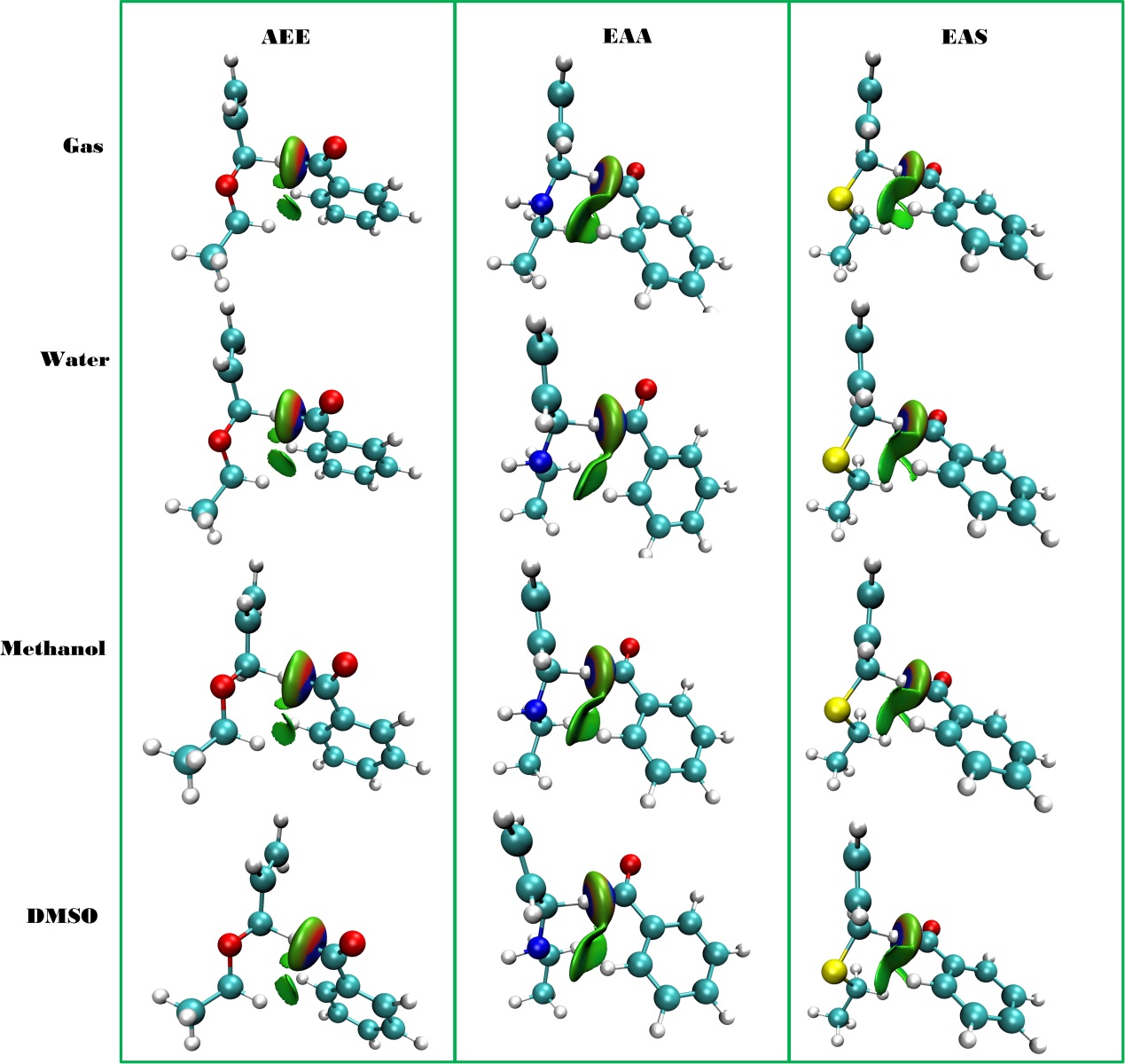
**

**Figure S4** The surface plots of vdW surface in green area from different solvents

**Table S1** The values for highest occupied molecular orbital (HOMO) and lowest unoccupied molecular orbital (LUMO) of the AEE, EAA and EAS at the M06-2X/6-311++g(d,p) level.

|  |  | **HOMO**  **(eV)** | **LUMO**  **(eV)** | **△*E*_HOMO-LUMO_**  **(eV)** | **△*E*_HOMO-LUMO_**  **(kcal/mol)** |
| --- | --- | --- | --- | --- | --- |
| GAS | AEE | -8.77 | -0.01 | 8.76 | 202.01 |
|  | EAA | -7.94 | -0.03 | 7.91 | 182.41 |
|  | EAS | -7.51 | -0.05 | 7.46 | 172.03 |
| WATER | AEE | -8.86 | 0.17 | 9.03 | 208.11 |
|  | EAA | -8.06 | 0.16 | 8.23 | 189.79 |
|  | EAS | -7.50 | 0.15 | 7.65 | 176.41 |
| METH | AEE | -8.85 | 0.17 | 9.01 | 207.78 |
|  | EAA | -8.00 | 0.16 | 8.16 | 188.17 |
|  | EAS | -7.50 | 0.15 | 7.65 | 176.41 |
| DMSO | AEE | -8.72 | 0.15 | 8.87 | 204.55 |
|  | EAA | -7.97 | 0.16 | 8.14 | 187.71 |
|  | EAS | -7.50 | 0.15 | 7.65 | 176.41 |

**Table S2** The calculated quantum chemical parameters of title compounds.

| Compound | solvent | ionization energies (I) | electron affinities (A) | Electronega  -tivity (χ) | chemical hardness (η) | chemical softness (ζ) | chemical potential (μ) | Electrophi-licity index (ω) |
| --- | --- | --- | --- | --- | --- | --- | --- | --- |
| AEE | Gas | 8.7730 | 0.0130 | 4.3930 | 4.3800 | 0.1142 | -4.3930 | 2.2030 |
|  | Water | 8.8576 | -0.1699 | 4.3439 | 4.5138 | 0.1108 | -4.3439 | 2.0902 |
|  | Methanol | 8.8452 | -0.1680 | 4.3386 | 4.5066 | 0.1109 | -4.3386 | 2.0884 |
|  | DMSO | 8.7157 | -0.1515 | 4.2821 | 4.4336 | 0.1128 | -4.2821 | 2.0679 |
| EAA | Gas | 7.9384 | 0.0258 | 3.9821 | 3.9563 | 0.1264 | -3.9821 | 2.0040 |
|  | Water | 8.0634 | -0.1641 | 3.9497 | 4.1137 | 0.1215 | -3.9497 | 1.8961 |
|  | Methanol | 7.9962 | -0.1614 | 3.9174 | 4.0788 | 0.1226 | -3.9174 | 1.8812 |
|  | DMSO | 7.9727 | -0.1633 | 3.9047 | 4.0680 | 0.1229 | -3.9047 | 1.8740 |
| EAS | Gas | 7.5090 | 0.0508 | 3.7799 | 3.7291 | 0.1341 | -3.7799 | 1.9157 |
|  | Water | 7.5035 | -0.1504 | 3.6766 | 3.8269 | 0.1307 | -3.6766 | 1.7660 |
|  | Methanol | 7.5027 | -0.1465 | 3.6781 | 3.8246 | 0.1307 | -3.6781 | 1.7686 |
|  | DMSO | 7.5033 | -0.1485 | 3.6774 | 3.8259 | 0.1307 | -3.6774 | 1.7674 |

Study of the compounds through parameters such as ionization energies (I), electron affinities (A), electronegativity ( χ ),chemical hardness ( η ), chemical softness ( ζ ), chemical potential ( μ ), and the global electrophilicity index ( ω ) by Koopmans’s theory. It allows us to further understand the properties of various aspects. The calculation equation of each parameter is as follows:

 (1)

 (2)

$\eta=\frac{I-A}{2}$ (3)

$\chi=\frac{I+A}{2}$ (4)

$\mu=-\frac{I+A}{2}$ (5)

 (6)

$\zeta=\frac{1}{2\eta}$ (7)

The parameters are shown in **Table S2**. The chemical hardness ( η ) is an index of the thermodynamic stability of the compound and the electronegativity ( χ ) describes the ability of a molecule or functional group to attract electrons to itself. In order to measure of the stability of the system after the extra electrons it receives from the environment, which is represented by the electrophilicity index (ω).

**Table S3** Changes in bond angles and distances between the reaction complex (RC) and the transition state (TS).

|  | **Solvent** | **The minma energy**  **of surface(kcal/mol)** | **The maxmia energy**  **of surface(kcal/mol)** |
| --- | --- | --- | --- |
| AEE | GAS | -35.15 | 14.29 |
|  | WATER | -46.35 | 17.38 |
|  | METH | -45.78 | 17.23 |
| EAA | DMSO | -41.42 | 16.66 |
|  | GAS | -33.39 | 21.47 |
|  | WATER | -40.97 | 25.36 |
|  | METH | -39.87 | 25.13 |
|  | DMSO | -39.65 | 25.18 |
|  | GAS | -25.29 | 15.63 |
| EAS | WATER | -31.14 | 20.91 |
|  | METH | -30.91 | 20.70 |
|  | DMSO | -31.03 | 20.81 |

**Table S4** The rate constants value with and without tunneling correction as a function of temperature from 500 K to 2500 K.

**With tunneling correction**

|  |  | 500k | 1000k | 1500k | 2000k 2500k | | |
| --- | --- | --- | --- | --- | --- | --- | --- |
| AEE | Gas | 5.93E-18 | 2.98 E-15 | 5.1 E-14 | 2.89 E-13 | 9.71 E-13 |  |
|  | Water | 3.82E-17 | 7.76E-15 | 9.74E-14 | 4.72E-13 | 1.45E-12 |  |
|  | Methanol | 4.24E-17 | 1.28E-14 | 1.85E-13 | 9.61E-13 | 3.07E-12 |  |
|  | DMSO | 1.04E-17 | 3.18E-15 | 4.59E-14 | 2.38E-13 | 7.62E-13 |  |
| EAA | Gas | 1.41E-17 | 2.97E-15 | 3.77E-14 | 1.84E-13 | 5.67E-13 |  |
|  | Water | 3.14E-17 | 9.72E-15 | 1.43E-13 | 7.56E-13 | 2.44E-12 |  |
|  | Methanol | 8.99E-18 | 2.78E-15 | 4.07E-14 | 2.14E-13 | 6.90E-13 |  |
|  | DMSO | 6.81E-18 | 2.08E-15 | 3.03E-14 | 1.59E-13 | 5.11E-13 |  |
| EAS | Gas | 1.74E-17 | 2.97E-15 | 3.47E-14 | 1.62E-13 | 4.85E-13 |  |
|  | Water | 4.29E-17 | 5.94E-15 | 6.48E-14 | 2.93E-13 | 8.60E-13 |  |
|  | Methanol | 2.86E-17 | 6.21E-15 | 7.90E-14 | 3.87E-13 | 1.19E-12 |  |
|  | DMSO | 8.13E-18 | 2.04E-15 | 2.72E-14 | 1.36E-13 | 4.25E-13 |  |

**Without tunneling correction**

|  |  | 500k | 1000k | 1500k | 2000k 2500k | | |
| --- | --- | --- | --- | --- | --- | --- | --- |
| AEE | Gas | 2.09E-18 | 2.29 E-15 | 4.50E-14 | 2.68E-13 | 9.24 E-13 |  |
|  | Water | 1.35E-17 | 5.93E-15 | 8.58E-14 | 4.38E-13 | 1.37E-12 |  |
|  | Methanol | 1.49E-17 | 9.81E-15 | 1.63E-13 | 8.91E-13 | 2.92E-12 |  |
|  | DMSO | 3.66E-18 | 2.43E-15 | 4.05E-14 | 2.21E-13 | 7.24E-13 |  |
| EAA | Gas | 5.10E-18 | 2.29E-15 | 3.33E-14 | 1.71E-13 | 5.39E-13 |  |
|  | Water | 1.10E-17 | 7.46E-15 | 1.26E-13 | 7.02E-13 | 2.32E-12 |  |
|  | Methanol | 3.13E-18 | 2.13E-15 | 3.60E-14 | 1.99E-13 | 6.57E-13 |  |
|  | DMSO | 2.40E-18 | 1.60E-15 | 2.68E-14 | 1.48E-13 | 4.86E-13 |  |
| EAS | Gas | 6.57E-18 | 2.30E-15 | 3.07E-14 | 1.50E-13 | 4.61E-13 |  |
|  | Water | 1.63E-17 | 4.60E-15 | 5.74E-14 | 2.72E-13 | 8.18E-13 |  |
|  | Methanol | 1.06E-17 | 4.81E-15 | 6.99E-14 | 3.60E-13 | 1.14E-12 |  |
|  | DMSO | 2.99E-18 | 1.58E-15 | 2.41E-14 | 1.27E-13 | 4.04E-13 |  |

**Table S5** The calculated quantum chemical Hirshfeld charges and Fukui Function of title compounds.

**AEE**

| Atom Number | q_(N)_ | q_(N+1)_ | q_(N-1)_ | f^-^ | f^+^ | f^0^ |
| --- | --- | --- | --- | --- | --- | --- |
| 1(H) | 0.0281 | 0.1292 | -0.0549 | -0.0830 | -0.1011 | -0.0921 |
| 2(C) | -0.0359 | 0.0807 | -0.0556 | -0.0196 | -0.1167 | -0.0682 |
| 3(H) | 0.0430 | 0.1010 | -0.0125 | -0.0555 | -0.0580 | -0.0568 |
| 4(C) | -0.0849 | 0.0957 | -0.1306 | -0.0457 | -0.1806 | -0.1131 |
| 5(H) | 0.0372 | 0.0942 | -0.0461 | -0.0833 | -0.0570 | -0.0701 |
| 6(H) | 0.0407 | 0.1052 | -0.0410 | -0.0816 | -0.0646 | -0.0731 |
| 7(C) | 0.0231 | 0.0802 | 0.0035 | -0.0196 | -0.0571 | -0.0383 |
| 8(H) | 0.0291 | 0.0977 | -0.0329 | -0.0621 | -0.0685 | -0.0653 |
| 9(C) | 0.0256 | 0.0582 | 0.0018 | -0.0239 | -0.0326 | -0.0282 |
| 10(H) | 0.0245 | 0.0600 | -0.0614 | -0.0859 | -0.0354 | -0.0607 |
| 11(H | 0.0239 | 0.0560 | -0.0654 | -0.0893 | -0.0321 | -0.0607 |
| 12(C) | -0.0878 | -0.0701 | -0.1185 | -0.0306 | -0.0177 | -0.0242 |
| 13(H) | 0.0351 | 0.0632 | -0.0633 | -0.0983 | -0.0281 | -0.0632 |
| 14(H) | 0.0348 | 0.0525 | -0.0265 | -0.0613 | -0.0176 | -0.0395 |
| 15(H) | 0.0355 | 0.0543 | -0.0239 | -0.0594 | -0.0188 | -0.0391 |
| 16(O) | -0.1718 | -0.0581 | -0.1795 | -0.0077 | -0.1137 | -0.0607 |

Nucleophilic attack **:** f^+^= q_(N)_- q_(N+1)_ Electrophilic attack : f^-^= q_(N-1)_-q_(N)_  Radical attack: f^0^= (q_(N-1)_- q_(N+1)_)/2

Hirshfeld charges in different solvents

| Atom Number | Gas | Water | Methanol | DMSO |
| --- | --- | --- | --- | --- |
| 1(H) | 0.02814294 | 0.04090813 | 0.04039056 | 0.03673941 |
| 2(C) | -0.03596444 | -0.05073711 | -0.05018062 | -0.04750325 |
| 3(H) | 0.04298982 | 0.04495155 | 0.04487468 | 0.044155 |
| 4(C) | -0.08488662 | -0.0894106 | -0.08960514 | -0.09364883 |
| 5(H) | 0.0371631 | 0.04629822 | 0.04593112 | 0.04455101 |
| 6(H) | 0.0406498 | 0.04719619 | 0.04690815 | 0.04548339 |
| 7(C) | 0.02308439 | 0.01961334 | 0.01980172 | 0.02022451 |
| 8(H) | 0.02911346 | 0.04064166 | 0.04020624 | 0.0369024 |
| 9(C) | 0.02562509 | 0.02494092 | 0.02497095 | 0.02532424 |
| 10(H) | 0.02454101 | 0.035883 | 0.03541211 | 0.03168364 |
| 11(H | 0.02390448 | 0.03564857 | 0.0351371 | 0.03145984 |
| 12(C) | -0.08784996 | -0.09305433 | -0.09288245 | -0.09201435 |
| 13(H) | 0.03506623 | 0.04401065 | 0.04350884 | 0.04146417 |
| 14(H) | 0.03483803 | 0.03329216 | 0.03336988 | 0.03247035 |
| 15(H) | 0.03544711 | 0.03326603 | 0.03337837 | 0.0324604 |
| 16(O) | -0.17186443 | -0.21344836 | -0.21122151 | -0.18975193 |

**EAA**

| Atom Number | q_(N)_ | q_(N+1)_ | q_(N-1)_ | f^-^ | f^+^ | f^0^ |
| --- | --- | --- | --- | --- | --- | --- |
| 1(H ) | 0.0348 | 0.0777 | -0.0116 | -0.0465 | -0.0429 | -0.0447 |
| 2(C ) | -0.0365 | -0.0258 | -0.0533 | -0.0167 | -0.0107 | -0.0137 |
| 3(H ) | 0.0390 | 0.0619 | -0.0100 | -0.0489 | -0.0229 | -0.0359 |
| 4(C ) | -0.0911 | -0.0112 | -0.1347 | -0.0436 | -0.0800 | -0.0618 |
| 5(H ) | 0.0347 | 0.0681 | -0.0594 | -0.0941 | -0.0334 | -0.0638 |
| 6(H ) | 0.0384 | 0.0804 | -0.0375 | -0.0759 | -0.0420 | -0.0590 |
| 7(C ) | -0.0114 | 0.0341 | -0.0271 | -0.0157 | -0.0455 | -0.0306 |
| 8(H ) | 0.0198 | 0.0824 | -0.0460 | -0.0658 | -0.0626 | -0.0642 |
| 9(C ) | -0.0097 | 0.0386 | -0.0298 | -0.0202 | -0.0483 | -0.0342 |
| 10(H ) | 0.0147 | 0.0860 | -0.0765 | -0.0911 | -0.0713 | -0.0812 |
| 11(H ) | 0.0297 | 0.0811 | -0.0219 | -0.0515 | -0.0514 | -0.0515 |
| 12(C ) | -0.0886 | -0.0639 | -0.1201 | -0.0315 | -0.0247 | -0.0281 |
| 13(H ) | 0.0334 | 0.0723 | -0.0490 | -0.0825 | -0.0389 | -0.0607 |
| 14(H ) | 0.0315 | 0.0561 | -0.0229 | -0.0544 | -0.0246 | -0.0395 |
| 15(H ) | 0.0289 | 0.0568 | -0.0631 | -0.0920 | -0.0279 | -0.0600 |
| 16(N ) | -0.1563 | 0.1253 | -0.1650 | -0.0087 | -0.2816 | -0.1452 |
| 17(H ) | 0.0891 | 0.1802 | 0.0182 | -0.0709 | -0.0910 | -0.0810 |

Nucleophilic attack **:** f^+^= q_(N)_- q_(N+1)_ Electrophilic attack : f^-^= q_(N-1)_-q_(N)_  Radical attack: f^0^= (q_(N-1)_- q_(N+1)_)/2

Hirshfeld charges in different solvents

| Atom Number | Gas | Water | Methanol | DMSO |
| --- | --- | --- | --- | --- |
| 1(H ) | 0.03483215 | 0.03910432 | 0.03922524 | 0.03936403 |
| 2(C ) | -0.03659309 | -0.04615674 | -0.0458913 | -0.04615442 |
| 3(H ) | 0.03896407 | 0.03884535 | 0.03847105 | 0.03821367 |
| 4(C ) | -0.09119093 | -0.09969035 | -0.10043167 | -0.10073968 |
| 5(H ) | 0.03468964 | 0.04192623 | 0.04147431 | 0.04159056 |
| 6(H ) | 0.03841315 | 0.04308703 | 0.04278949 | 0.04282246 |
| 7(C ) | -0.0114384 | -0.01432018 | -0.01400771 | -0.01401053 |
| 8(H ) | 0.01975301 | 0.02954111 | 0.02891469 | 0.0290428 |
| 9(C ) | -0.0096956 | -0.0107198 | -0.01070342 | -0.01071894 |
| 10(H ) | 0.01467584 | 0.02336727 | 0.02252274 | 0.02257016 |
| 11(H ) | 0.02965009 | 0.03210226 | 0.03233292 | 0.03242563 |
| 12(C ) | -0.08861052 | -0.09256271 | -0.09269136 | -0.09289368 |
| 13(H ) | 0.03343049 | 0.03774727 | 0.03724767 | 0.03719134 |
| 14(H ) | 0.03147625 | 0.02664306 | 0.02650992 | 0.02616357 |
| 15(H ) | 0.02886229 | 0.03386266 | 0.0334436 | 0.03353067 |
| 16(N ) | -0.15636074 | -0.17936494 | -0.17618257 | -0.17561863 |
| 17(H ) | 0.08914232 | 0.09658815 | 0.09697639 | 0.097221 |

**EAS**

| Atom Number | q_(N)_ | q_(N+1)_ | q_(N-1)_ | f^-^ | f^+^ | f^0^ |
| --- | --- | --- | --- | --- | --- | --- |
| 1(H ) | 0.0376 | 0.0778 | -0.0389 | -0.0765 | -0.0402 | -0.0584 |
| 2(C ) | -0.0384 | -0.0413 | -0.0452 | -0.0068 | 0.0029 | -0.0020 |
| 3(H ) | 0.0399 | 0.0604 | -0.0007 | -0.0406 | -0.0204 | -0.0305 |
| 4(C ) | -0.0838 | -0.0198 | -0.1122 | -0.0283 | -0.0640 | -0.0462 |
| 5(H ) | 0.0388 | 0.0633 | 0.0016 | -0.0372 | -0.0245 | -0.0308 |
| 6(H ) | 0.0414 | 0.0767 | 0.0151 | -0.0263 | -0.0353 | -0.0308 |
| 7(C ) | -0.0504 | -0.0093 | -0.0685 | -0.0180 | -0.0412 | -0.0296 |
| 8(H ) | 0.0358 | 0.0828 | -0.0291 | -0.0649 | -0.0470 | -0.0560 |
| 9(C ) | -0.0485 | -0.0073 | -0.0790 | -0.0305 | -0.0412 | -0.0358 |
| 10(H ) | 0.0324 | 0.0771 | -0.0789 | -0.1114 | -0.0447 | -0.0780 |
| 11(H ) | 0.0316 | 0.0817 | -0.0844 | -0.1159 | -0.0502 | -0.0830 |
| 12(C ) | -0.0855 | -0.0627 | -0.1250 | -0.0395 | -0.0228 | -0.0311 |
| 13(H ) | 0.0361 | 0.0717 | -0.0934 | -0.1295 | -0.0355 | -0.0825 |
| 14(H ) | 0.0333 | 0.0579 | -0.0347 | -0.0680 | -0.0246 | -0.0463 |
| 15(H ) | 0.0334 | 0.0569 | -0.0342 | -0.0676 | -0.0235 | -0.0456 |
| 16(S ) | -0.0536 | 0.4341 | -0.1179 | -0.0644 | -0.4877 | -0.2760 |

Nucleophilic attack **:** f^+^= q_(N)_- q_(N+1)_ Electrophilic attack : f^-^= q_(N-1)_-q_(N)_  Radical attack: f^0^= (q_(N-1)_- q_(N+1)_)/2

Hirshfeld charges in different solvents

| Atom Number | Gas | Water | Methanol | DMSO |
| --- | --- | --- | --- | --- |
| 1(H ) | 0.03759753 | 0.048425 | 0.048114 | 0.0482867 |
| 2(C ) | -0.03839793 | -0.04617 | -0.04594 | -0.04606627 |
| 3(H ) | 0.03993721 | 0.043752 | 0.043706 | 0.04373636 |
| 4(C ) | -0.08385914 | -0.0949 | -0.09448 | -0.09469072 |
| 5(H ) | 0.03882936 | 0.045001 | 0.044815 | 0.04491705 |
| 6(H ) | 0.04138636 | 0.045854 | 0.045735 | 0.0457926 |
| 7(C ) | -0.05044352 | -0.0489 | -0.04891 | -0.04889641 |
| 8(H ) | 0.03578481 | 0.044869 | 0.044398 | 0.04461757 |
| 9(C ) | -0.04851662 | -0.04368 | -0.04393 | -0.04381215 |
| 10(H ) | 0.03243048 | 0.041254 | 0.04103 | 0.04116208 |
| 11(H ) | 0.03155733 | 0.041112 | 0.040644 | 0.04087652 |
| 12(C ) | -0.08549616 | -0.08768 | -0.08754 | -0.08760248 |
| 13(H ) | 0.03612819 | 0.042543 | 0.042321 | 0.04243622 |
| 14(H ) | 0.03327789 | 0.032625 | 0.032762 | 0.03269888 |
| 15(H ) | 0.03339472 | 0.032575 | 0.032626 | 0.03259411 |
| 16(S ) | -0.05361051 | -0.09667 | -0.09534 | -0.09605006 |

### 3. The definitions of abbreviation

**01. Deformation energy (E_d_):**

The E_d_ is the energy required for the geometrical deformations of the fragments from a reference geometry (often, but again not necessarily, their equilibrium geometry) to the geometry they acquire at the transition state. It is therefore strongly related to the structural rigidity of the fragments. As the reference geometries are usually not distorted, this term is typically destabilizing. In principle, the strain term can also incorporate excitations to electronic configurations that are better suited or required for the interaction studied, but most often the reference fragments are chosen as already having the correct valence configuration.

**02. Interaction energy (E_i_):**

Total energy variation due to interfragment interaction between fragments A and B may be expressed as (fragments mentioned below are in complex structure, so deformation energy due to distortion of fragment structure during combination is not taken into account in this context):

∆𝐸 _int_ = 𝐸_𝐴𝐵_ − 𝐸_𝐴_ − 𝐸_𝐵_

where E_AB_ is the complex electronic energy, E _A_ and E _B_ are electronic energies of A and B at complex geometry.
**03. Activation energy (E_a_):**

The E_a_ is the height of a reaction barrier. This is done by splitting its energy at this point, ΔE_a_, into the strain energy term ΔE_d_, and the interaction energy term ΔE_int_: ΔE_a_ = ΔE_d_ + ΔE_int_. (1) The strain energy ΔE_d_ is the energy required for the geometrical deformations of the fragments from a reference geometry (often, but again not necessarily, their equilibrium geometry) to the geometry they acquire at the transition state.
**04. Electrostatic potential (ESP):**

In a molecular system, the electrostatic potential (ESP) can be written as:

where Z and R denote nuclear charge and nuclear position, respectively. ESP measures the electrostatic interaction between a unit point charge placed at r and the system of interest. A positive (negative) value implies that current position is dominated by nuclear (electronic) charges.
**05. Localized Orbital Locator (LOL):**

The present localized orbital locator (LOL) clearly displays the locations of the classic VSEPR electronic groups.
**06. Electron Localization Function (ELF):**
ELF depends on the excess kinetic energy D(r) caused by Pauli repulsion and the Thomas-Fermi kinetic energy density D_0_(r). So that ELF can be expressed as


and its value ranges from 0 to 1.

**Reference**

1. WIREs. Comput. Mol. Sci. 2015, 5:324–343.
2. Angew. Chem. Int. Ed. 2017, 56, 10070 – 10086.
3. J. Mol. Graph. Model. 2012,38, 314–323.
4. WIREs. Comput. Mol. Sci. 2011, 1, 153–163.
5. J. Mol. Struct. 2000, 527, 51-61.
6. J. Mol. Struct. 2020, 1201,127208,
